# Health Benefits of Resveratrol in Kidney Disease: Evidence from In Vitro and In Vivo Studies

**DOI:** 10.3390/nu11071624

**Published:** 2019-07-17

**Authors:** Danja J. Den Hartogh, Evangelia Tsiani

**Affiliations:** 1 Department of Health Sciences, Brock University, St. Catharines, ON L2S 3A1, Canada; 2 Centre for Bone and Muscle Health, Brock University, St. Catharines, ON L2S 3A1, Canada

**Keywords:** resveratrol, kidney disease, mesangial cells, renal epithelial cells, fibroblasts, glomerulosclerosis, renal cancer

## Abstract

Different diseases and disorders that affect the kidneys include, but are not limited to, glomerulonephritis, diabetic nephropathy, polycystic kidney disease, kidney stones, renal fibrosis, sepsis, and renal cell carcinoma. Kidney disease tends to develop over many years, making it difficult to identify until much later when kidney function is severely impaired and undergoing kidney failure. Although conservative care, symptom management, medication, dialysis, transplantation, and aggressive renal cancer therapy are some of the current strategies/approaches to kidney disease treatment, new preventative targeted therapies are needed. Epidemiological studies have suggested that a diet rich in fruits and vegetables is associated with health benefits including protection against kidney disease and renal cancer. Resveratrol, a polyphenol found in grapes and berries, has been reported to have antioxidant, anti-inflammatory, antidiabetic, hepatoprotective, neuroprotective, and anti-cancer properties. The current review summarizes the existing in vitro and in vivo animal and human studies examining the nephroprotective effects of resveratrol.

## 1. Introduction

### 1.1. Kidney Function in Health and Disease

**Figure 1:**
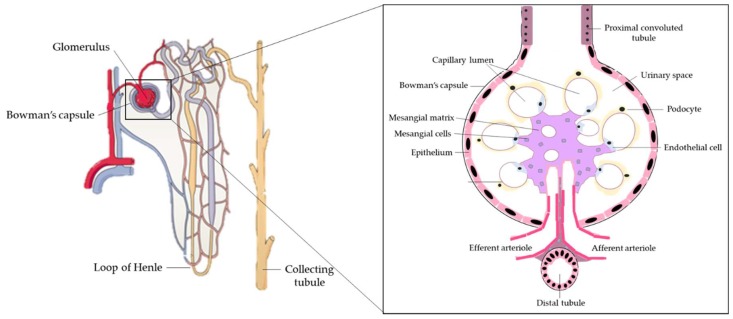
The structure of the glomerulus and nephron.

The kidneys are a pair of organs located below and posterior to the liver in the peritoneal cavity whose main function is blood filtration and salt and water homeostasis [1]. The kidney is divided into three regions: the outer cortex, medulla, and inner hilum. The renal cortex contains the functional unit of the kidney known as the nephron, with approximately one million nephrons located within each kidney (Figure 1) [2]. Each nephron is responsible for filtration as blood enters the kidney, which migrates through the length of the nephron where specialized regions reabsorb water and small molecules before it is secreted as urine. The nephron can be further divided into the renal corpuscle (Bowman’s capsule) and renal tubule [2]. Located within Bowman’s capsule is the glomerulus, a filtering unit of blood vessels which is responsible for the majority of filtration within the kidney. Throughout all these structures, the kidney is connected to a highly vascularized network of arteries, veins, and nerves, entering and exiting at the renal hilum [2]. In addition to filtration and reabsorption, the kidneys also produce hormones such as renin, erythropoietin, and calcitriol/vitamin D_3_, that regulate blood pressure, help control red blood cell production, and maintain bone metabolism and health [3,4]. 

The kidneys are highly metabolic organs that consume roughly 7% of the total oxygen available for function and filter roughly 25% of the cardiac output, amounting to 114 to 142 L of blood per day [5,6]. The kidneys filter and reabsorb blood through a three-step process. Initially, within the nephron, the renal corpuscle filters molecules, including glucose, proteins, ions, and urea, before the filtrate continues to the proximal convoluted tubule [7]. Glomerular mesangial cells occupy a fundamental position in the renal glomerulus, forming the central tuft-like structure of the glomerular microvasculature involved in the generation of inflammatory mediators and extracellular matrix (ECM) proteins, They are also responsible for contractile function. Mesangial cells contract or relax to modify glomerular filtration locally in response to vasoconstrictive or vasorelaxant agents, respectively [8]. In addition, renal podocytes are cells that wrap around the capillaries of the glomerulus in the Bowman’s capsule. Functionally, podocytes, together with renal endothelial cells, form the filtration barrier and interact with mesangial cells to regulate glomeruli function [9]. Kidney fibroblasts are found in the interstitium, are involved in the production of ECM components such as glycosaminoglycans and collagen, and act to maintain ECM homeostasis by producing ECM-degrading proteases. With dysfunction, fibroblasts continue to produce ECM components, resulting in tubulointerstitial fibrosis and renal failure [10]. 

As the blood continues to pass through the Bowman’s capsule, molecules including urea, uric acid, and creatinine are absorbed in the proximal convoluted tubule of the nephron by tubular reabsorption [11]. Renal epithelial cells, found in the outer layer of the renal tubule, are primarily involved in blood clearance and glomerular filtration via active transport. Renal dysfunction and necrosis associated with renal failure occur when renal epithelial cells are injured [12]. From the renal tubules, the blood passes through the Loop of Henle, a long U-shaped tubule that reabsorbs water and sodium chloride and further concentrates the filtrate [11]. The Loop of Henle then becomes the distal convoluted tubule, which reabsorbs calcium, sodium, and chloride and is responsible for pH regulation of the urine by bicarbonate absorption [13]. Any molecules/substrates including water not reabsorbed through this process will be eliminated in the urine, with roughly 1–2 L of urine produced per day [7]. 

A global assessment of renal function is often ascertained by the glomerular filtration rate (GFR) [14]. GFR is calculated based on serum creatinine, age, body weight, and gender, and is used clinically to evaluate the level of kidney function and disease progression [15]. Chronic kidney disease can be separated into five stages based on GFR. Stage 1 is defined by a GFR of 90 mL/min/m^2^ or higher, depicting 90–100% kidney function, while stage 5, or end-stage kidney failure, is defined by a GFR of less than 15 mL/min/m^2^, depicting less than 15% kidney function [14]. When the kidneys are damaged, their function becomes impaired and the GFR is reduced, resulting in acid/base (pH) balance dysregulation, electrolyte imbalance, malabsorption, and health problems, including osteoporosis, nerve damage, and others [16,17,18].

Chronic kidney disease (CKD) is defined as kidney damage, or decreased kidney function present for longer than three months. In addition, CKD requires an estimated GFR of less than 34.68 mL/min/m^2^ and abnormalities in biopsy/renal imaging results [19]. It is estimated that 1.3 million to 2.9 million Canadians have CKD, resulting in a significant economic burden on the Canadian health care system [20]. In the United States of America, in 2015, health care expenditures on chronic and end-stage kidney disease totaled more than 64 billion and 34 billion U.S. dollars, respectively, highlighting the significant burden kidney disease exerts on the health care system [21]. 

Kidney disease tends to develop over many years, making it difficult to identify until much later when kidney function is severely impaired. Physiologically, CKD arises due to many pathological injuries that destroys some of the nephrons, resulting in the nephrons overcompensating by hyperfiltration. Over time, glomerular hypertension, albuminuria, and loss of renal function develop [22]. The increase in glomerular capillary pressure leads to glomerular capillary wall destruction, dysfunction of podocytes that cover the capillaries, and increased macromolecule permeability [22,23]. In conjunction, increased pro-inflammatory mediators are released that stimulate the proliferation of fibrotic cells. In addition, accumulation of ECM molecules results in scar formation and renal failure [22,23,24]. Currently, treatment strategies exist for CKD, with all options aimed at relieving or preventing the condition from worsening, including conservative care, medication, dialysis, or transplantation [25,26]. 

CKD is not the only form of kidney disorder that can severely affect an individual, with many other disorders severely afflicting the kidney and renal system, such as polycystic kidney disease (PKD), a genetic disorder, either autosomal dominant or recessive, characterized by cyst formation in the kidneys [27]. This causes the kidneys to have a rough surface and increased inflammation [27]. Glomerulonephritis is term used to describe a range of immune-mediated disorders resulting in inflammation of the glomerulus and other regions of the kidney [28]. The inflammation within the kidney disrupts blood filtration, leading to decreased urination, high blood pressure, hematuria, and albuminuria [28]. 

Diabetic nephropathy characterized by glomeruli damage and impaired blood filtration develops in more than 50% of people with type 2 diabetes mellitus (T2DM) [29]. These diseases can ultimately result in end-stage renal disease, or kidney failure, where the kidneys have completely stopped working, requiring dialysis or a kidney transplant for maintenance of life.

Renal cell carcinoma (RCC), also known as cancer of the kidney, is the sixth and tenth most common cancer in men and women, respectively, accounting for more than 140,000 deaths yearly and ranking as the 13th most common cause of cancer death worldwide [30,31]. RCC originates in the lining of the proximal convoluted tubule and encompasses approximately ninety percent of all kidney cancer cases in adults [32]. RCC is characterized by decreased kidney filtration, anemia, and increased blood pressure, resulting in complete kidney failure [33]. Current treatment strategies of RCC include surgery (partial or radical nephrectomy), chemotherapy, immunotherapy, and radiation therapy [34]. 

Epidemiological studies have suggested that diets rich in fruits and vegetables help regulate body weight and protect against chronic diseases including cardiovascular disease, diabetes mellitus, cancer and kidney disease [35,36,37]. Specific components, known as polyphenols, show potential health benefits and preventative and therapeutic properties against chronic diseases, including kidney disease [38,39,40,41]. 

### 1.2. Resveratrol

Resveratrol (RSV) (3,5,4′-trihydroxy-trans-stillbene) is a polyphenol belonging to the family of stilbenes, based on shared common structure of two phenyl moieties connected by a two-carbon methylene bridge [42]. RSV is found in the skin of grapes, in berries, and peanuts, with considerably high levels in grape juice (0.19–0.96 mg/L), and red wine (1.9 ± 1.7 mg/L) [43,44,45]. RSV has been studied for its pharmacological effects, including antioxidant, anti-inflammatory, immunomodulatory, hepatoprotective, anti-cancer, anti-atherosclerotic, and anti-diabetic properties [42,46,47,48,49,50,51]. 

The bioavailability of RSV is relatively low due to its low absorption, rapid metabolism, and elimination. A number of past reviews have focused on resveratrol’s bioavailability [52,53,54] and interested readers are recommended to consult these reviews [52,53,54]. Initial studies in humans, showed low levels of unmetabolized RSV in the plasma upon a single oral administration dose of 5 to 25 mg [54,55,56]. Administration of 25 mg trans-RSV resulted in total resveratrol peak blood concentration of 1.8–2 µM after 60 min [56]. This was similar to another study which showed that increasing doses (500 mg to 5000 mg) of oral administered trans-RSV resulted in plasma levels of 0.3–2.3 µM within 50–90 min [55].

A number of studies have examined tissue distribution of RSV [57,58,59]. Oral administration of ^3^H-trans-resveratrol in rats resulted in detectable RSV levels in plasma and different tissues with liver and kidney having the highest concentration [57]. Similarly, a single intravenous administration of *trans*-RSV (15 mg/kg body weight (b.w.)) in rats resulted in significant serum levels (0.025 µM), and examination of tissue distribution found the highest levels in the kidney (1.45 nmol/g) and lung (1.13 nmol/g) [58].

Once absorbed, RSV is metabolized by conjugation to glucuronic acid or sulfate to form glucuronides, sulfates, or sulfoglucuronides, with sulfate being the major metabolite found in plasma and urine [60]. Intragastric administration of RSV (472 mg) in pigs followed by examination of RSV and its metabolites showed dihydroresveratrol (DH-RSV) and RSV-3-*O*-glucuronide as the main metabolites [59]. Limited studies exist in humans investigating the bioavailability and metabolism of RSV, and more studies are needed.

Overall, although the bioavailability of RSV may be low, studies still found plasma levels of RSV and its metabolites in the micromolar range [55,61]. Many in vitro studies have shown that various biological effects and modification of cell signaling pathways and gene expression occur with micromolar levels of RSV [55,61]. 

The current review is focused on the effects of resveratrol on kidney disease and all existing in vitro and in vivo animal and human studies are presented. The studies are presented chronologically, and in addition to the text, are organized and presented in a table format for easier reader access of the information. 

## 2. Resveratrol’s Effects on Kidney Disease 

### 2.1. In Vitro Studies: Effects of Resveratrol on Mesangial Cells

Glomerular mesangial cells occupy a central position in the renal glomerulus forming the central tuft-like structure of the glomerular microvasculature, involved in the generation of inflammatory mediators (such as cytokines, macromolecules and immune complexes), and are responsible for the contractile function. Mesangial cells contract or relax to modify glomerular filtration locally in response to vasoconstrictive or vasorelaxant agents, respectively [8]. Mesangial matrix expansion and vaso-mediator release result in decreased glomerular surface area and hemodynamics, reducing GFR. Mesangial cell function is affected by immunologic injury and metabolic disease, resulting in impaired filtration [62].

A study by Uchida et al. (2005), demonstrated potential pro-inflammatory effects of RSV (50–75 µM) treatment of glomerular mesangial cells [63]. Nuclear factor kappa light-chain-enhancer of activated B cells (NF-κB) are a family of transcription factors that once activated result in transcriptional activation of inflammatory and immunoregulatory genes. These cells are activated in individuals with glomerulonephritis [64]. Glomerular mesangial cells co-stimulated with pro-inflammatory cytokine interleukin-1 (IL-1) or tumor necrosis factor alpha (TNF-α) and RSV treatment resulted in increased NF-κB activity [63] (Table 1). This increased pro-inflammatory effect was also shown in kidney proximal tubule LLCPK1 cells. However, when glomerular mesangial cells and LLCPK1 cells were treated with RSV alone, no significant change in NF-κB occurred, suggesting that RSV exerts pro-inflammatory effects only in the presence of pre-existing pro-inflammatory cytokines [63]. 

Exposure of primary mesangial cells to 10 µM RSV resulted in significant inhibition of gentamicin-induced cell contraction, typically seen in gentamicin-induced nephrotoxicity [65]. The RSV effects were abolished by the nicotinamide adenine dinucleotide phosphate (NADP(H)) inhibitor diphenylene iodinium, indicating that NADP(H) is involved in the action of RSV (Table 1) [65].

In a study by Venkatesan et al. (2008), RSV treatment of mesangial cells significantly inhibited the platelet-derived growth factor (PDGF)-induced cell proliferation (Table 1) [66]. PDGF is a potent stimulus for mesangial cell proliferation and involved in the pathogenesis of glomerulonephritis [66]. RSV abolished the PDGF-induced tyrosine-751 and tyrosine-716 phosphorylation of the PDGF receptor, the binding sites for phosphoinositide 3-kinase (PI3K) and growth factor receptor-bound protein 2 (Grb2), respectively. The PDGF-stimulated PI3K, protein kinase B (Akt), extracellular signal-regulated kinases 1/2 (ERK1/2) and proto-oncogene tyrosine-protein kinase Src (c-Src) activity was significantly blocked by RSV treatment [66]. Importantly, RSV increased the activity of tyrosine-protein phosphatase non-receptor type 1B (PTP1B), the phosphatase responsible for dephosphorylation of PDGF receptor tyrosine-751 and tyrosine-716, indicating an effect of RSV on PDGF signaling at the receptor level [66]. 

Treatment of primary mesangial cells with RSV prior to high glucose exposure reduced the hyperglycemia-induced reactive oxygen species (ROS) production and mitochondrial superoxide generation, and increased manganese superoxide dismutase (MnSOD) activity (Table 1) [67]. RSV treatment prevented the decrease in mitochondrial complex III activity induced by high glucose, which is a major source of mitochondrial oxidative stress. Additionally, treatment with RSV restored the mitochondrial membrane potential hyperpolarization, increased adenosine triphosphate (ATP) production, and preserved mitochondrial DNA content [67]. Importantly, all the effects of RSV were blocked by sirtuin 1 (SIRT1) inhibition [67] clearly indicating an important role of SIRT1 in mediating the effects of RSV.

RSV treatment of CRL-2573 rat kidney cell line and primary mesangial cells prevented high glucose-induced mesangial cell proliferation and fibronectin expression (Table 1) [68]. In addition, treatment with RSV attenuated the high glucose-induced c-Jun N-terminal kinase (JNK) and NF-κB activation, and reduced NADPH oxidase activity and ROS production. Overall, RSV treatment abolished the hyperglycemia-induced oxidative stress [68].

Treatment of HBYZ-1 mesangial cells with RSV for 72 h significantly attenuated the high glucose-induced effects on adiponectin receptor 1 (AdipoR1) messenger ribonucleic acid (mRNA) and protein levels (Table 1) [69]. The increase in AdipoR1 mRNA and protein levels was abolished in the presence of forkhead box 01 (FOX01) short hairpin RNA (shRNA), indicating a role of FOX01 in the effects of RSV [69]. 

In a study by Xu et al. (2014), treatment with RSV reduced the high glucose-induced mesangial cell proliferation, phosphorylated Akt, and NF-κB p65 protein levels, as well as the protein level of the inflammation marker plasminogen activator inhibitor-1 (PAI-1) (Table 1) [70]. Inhibitors of Akt had the same effect on PAI-1 expression as RSV, suggesting that the effects of RSV may be mediated by Akt [70]. 

In another study, exposure of CRL-2573 mesangial cells to RSV resulted in the attenuation of the high glucose-induced cell proliferation (Table 1) [71]. In addition, RSV attenuated the high glucose-induced increase in p38 phosphorylation, transforming growth factor-β1 (TGF-β1), and fibronectin expression [71].

Treatment of SV40 MES 13 cells with RSV attenuated the TGF-β1-induced mitochondrial function, with increased mitochondrial membrane potential and ATP production, and reduced ROS production [72]. In addition, RSV increased mitochondrial complex I/III activities and fission/fusion (NDUFB8 and ATP β nuclear-encoded) protein levels [72], indicating a protective effect of RSV on the electron transport chain. RSV treatment also increased SIRT1 protein levels, while peroxisome proliferator-activated receptor gamma coactivator (PGC)-1α and acetylated-PGC-1α protein levels were reduced, suggesting that the mitochondrial protective effects of RSV may be associated with SIRT1 activation (Table 1) [72].

Overall, these studies show that the treatment of mesangial cells with RSV attenuated the basal, PDGF-, high glucose- and TGF-β1-induced cell proliferation. In addition, RSV treatment reduced the high glucose- and TGF-β1-induced oxidative stress and inflammation, reduced mitochondrial superoxide and ROS production, and increased MnSOD and mitochondrial complex III activity. The production of the extracellular matrix protein, fibronectin, was significantly inhibited by RSV treatment. RSV treatment significantly reduced the high glucose-induced effects by regulating NF-κB, JNK, Akt, and p38 signaling (Table 1).

### 2.2. In Vitro Studies: Effects of Resveratrol on Renal Epithelial Cells

Injury in renal epithelial cells results in renal dysfunction and necrosis associated with renal failure [12]. In a study by Lee et al. (2010), treatment of glomerular epithelial cells with RSV resulted in reduced *de novo* protein synthesis (Table 2) [73]. Under high glucose conditions, liver kinase B1 (LKB1), the kinase upstream of AMP-activated protein kinase (AMPK), is acetylated, resulting in decreased LKB1 activity. RSV treatment prevented the high glucose-induced acetylation of LKB1 resulting in increased LKB1 activity and increased downstream phosphorylation/activation of AMPK [73]. RSV treatment inhibited the high glucose-induced fibronectin content. Additionally, the high glucose-induced RNA cap binding protein eukaryotic translation initiation factor (eIF)4E phosphorylation and the increase in eIF4G, eukaryotic translation elongation factor 2 (eEF2), and ribosomal protein S6 kinase (p70S6K) protein levels were abolished with RSV treatment [73].

Treatment of mouse proximal tubular cells with RSV reduced the cisplatin-induced injury and apoptosis. RSV decreased p53(serine (S)379) acetylation, which was reversed with the co-treatment of the SIRT1 inhibitor EX537 and SIRT1siRNA, indicating that the effects of RSV are mediated by SIRT1 [74]. In addition, pro-apoptotic p53 upregulated modulator of apoptosis (PUMA)-α and caspase-3 protein levels were reduced, while the anti-apoptotic member B-cell lymphoma-extra-large (Bcl-xL), Bcl-2-associated X (Bax), and BCL2-antagonist/killer (Bak) protein levels were increased with RSV treatment. Bax translocation to the mitochondria was reduced with RSV treatment, suggesting that the attenuation of cisplatin-induced cell death by RSV is via the down-regulation of pro-apoptotic protein expression (Table 2) [74]. 

In a study by Hong et al. (2013), treatment of human renal epithelial cells (HRCs) with RSV resulted in significantly reduced oxalate-mediated colonization and hyaluronan protein level, suggesting decreased kidney stone formation [75]. Additionally, RSV treatment suppressed the mRNA levels of genes involved in kidney stone formation, NADPH oxidase subunits (p22 and p47), MCP-1, and osteopontin (Table 2) [75]. RSV treatment attenuated the oxalate-induced ROS and malondialdehyde production, and increased antioxidant enzyme (SOD, glutathione peroxidase (GPx), and catalase) activities. TGF-β1, TGF-β type I receptor (TGFR-I), and TGF-β type II receptor (TGFR-II) protein levels induced by oxalate were significantly reduced with RSV treatment [75].

RSV treatment of mouse cortical collecting duct (mpkCCDc14) cells dose- and time-dependently decreased sodium transport [76]. mpkCCDc14 cells transfected with a green fluorescent protein PIP3 reporter (GFP-AKT-PH) followed by RSV treatment resulted in increased redistribution of PIP3 away from the plasma membrane and decreased sodium transporter expression [76]. The data suggest an inhibition of the PI3K-Akt-induced translocation of sodium transporters by RSV. Additionally, AMPKα protein levels were persistently increased with RSV treatment (Table 2) [76].

In a study by Bai et al. (2014), treatment of NRK-52E cells with RSV resulted in significantly decreased TGF-β1-induced cell proliferation, epithelial-to-mesenchymal transition (EMT), and extracellular matrix (EM) protein synthesis [77]. TGF-β1 treatment enhanced α-smooth muscle actin (α-SMA) and type III collagen protein levels and TGF-β1R, fibronectin, and Col1α1 mRNA levels, all of which were attenuated with RSV treatment [77]. Hedgehog signaling promotes myofibroblast activation and tubular EMT, resulting in increased matrix deposition and fibrosis formation [69]. RSV treatment inhibited TGF-β1-induced hedgehog pathway (shh and Gli1) mRNA levels (Table 2) [77].

Treatment of HK-2 cells with RSV attenuated the high glucose-induced EMT [78]. RSV treatment significantly reduced intracellular ROS levels and NADPH oxidase 1 (NOX1) and NOX4 protein levels. The high glucose-induced ERK1/2 activity was also reduced with RSV treatment. These data suggest that the protective effects of RSV against high glucose-induced EMT may be regulated by the NADPH oxidase/ROS/ERK cascade (Table 2) [78]. 

In a study by Xiao et al. (2016), RSV treatment of HK-2 cells resulted in significantly reduced aristolochic acid- and TGF-β-induced β-catenin nuclear translocation and protein levels, suggesting reduced EMT [79]. RSV treatment increased E-cadherin and SIRT1 protein levels and decreased profibrotic matrix metalloproteinase (MMP)-7, α-smooth muscle actin (α-SMA), and collagen type I alpha 1 (COL1A1) mRNA and protein levels (Table 2) [79]. 

Treatment of HK-2 cells with RSV resulted in significantly decreased ioxitalamate-induced cytotoxicity (Table 2) [80]. RSV treatment reduced cytosolic DNA fragmentation and 8-hydroxy-2′-deoxyguanosine (8-OHdG) formation, a biomarker of oxidative DNA damage. In addition, RSV treatment increased anti-apoptotic Bcl-2 and survivin protein levels and caspase-3 activity, while apoptosis and ROS production were reduced, indicating that RSV treatment prevents ioxitalamate-induced nephropathy [80].

Treatment of an in vitro model of polycystic kidney disease, the OX161 human autosomal dominant PKD renal epithelial cells, with RSV resulted in a dose-dependent decrease in MCP-1, complement factor B (CFB) protein levels, and TNF-α protein level and activity, suggesting reduced inflammation (Table 2) [81]. Additionally, phosphorylated pro-inflammatory NF-κBp65, p105, and p50 protein levels were reduced with RSV treatment. RSV increased superoxide dismutase-2 (SOD2) protein level, indicating reduced oxidative stress. In addition, treatment of Madin–Darby canine kidney (MDCK) epithelial cells with RSV resulted in reduced cyst numbers [81]. 

Lipopolysaccharide (LPS) and tunicamycin combined treatment of HK-2 cells resulted in decreased cell viability and increased pro-inflammatory cytokine (TNF-α, IL-1β, and IL-6) mRNA and protein levels to create an in vitro model of sepsis [82]. RSV treatment abolished these responses. In addition, RSV treatment reduced p65, phospho-p65, and phospho-inositol-requiring enzyme 1 (IRE1) protein levels in LPS and tunicamycin-treated HK-2 cells [82]. These data suggest that the effects of RSV on LPS and tunicamycin-induced inflammation and sepsis are mediated by IRE1-p65 pathway inhibition (Table 2) [82]. 

Treatment of HK-2 cells with RSV resulted in the attenuation of the high glucose-induced oxidative stress through increased SOD activity and decreased malondialdehyde (MDA) and ROS levels [83]. In addition, high glucose-induced catalase (CAT) and SIRT1 protein levels and SIRT1 deacetylase activity were increased with RSV. The protein level of acetylated-FoxO3a was significantly increased in HK-2 cells cultured under high glucose conditions and was ameliorated with RSV treatment (Table 2) [83]. 

Treatment of TCMK-1 renal epithelial cells with RSV resulted in reduced cadmium-induced apoptosis and increased cell viability [84]. Mitochondrial PGC-1α and SOD2 mRNA expression, both important regulators of mitochondrial biogenesis, were increased in cadmium-induced TCMK-1 cells treated with RSV. Mitochondria ROS (mROS) generation was reduced and mitochondrial SIRT3 protein level and activity was increased with RSV treatment (Table 2) [84].

RSV (5–20 µM) treatment of HK-2 cells dose-dependently reduced the TGF-β-induced EMT (Table 2) [85]. SIRT1 and epithelial cell marker E-cadherin protein levels were increased, while levels of fibrotic markers, α-SMA and fibronectin, were decreased with RSV treatment. These results were abolished with the co-treatment of SIRT1 inhibitor, EX527, suggesting that the effects of RSV is mediated through Sirt1 signaling [85]. RSV treatment also decreased the phosphorylation of Smad3 and reduced SIRT1 binding to Smad3 and Smad4, an important interaction involved in the progression of renal fibrosis [85]. High-dosage RSV treatment (40 µM), however, increased mitochondrial oxidative ROS production, fibronectin, α-SMA, snail, and pro-apoptotic Bax protein levels, while anti-apoptotic Bcl-2 protein levels were reduced, indicating an increased cytotoxicity and fibrotic phenotype. In addition, mitochondria length and density, ATP production, and mitochondrial biogenesis protein (PGC-1α, and mitochondrial transcription factor A (TFAM)) levels were reduced with high-dose RSV treatment, indicating mitochondrial dysfunction [85]. Therefore, low concentrations of RSV attenuate, while high concentrations of RSV mediate the oxidative stress and fibrotic effects in HK-2 cells.

Overall, these studies suggest that the treatment of renal epithelial cells with RSV attenuated the cisplatin-, high glucose-, oxalate- and TGF-β1-induced oxidative stress, reduced mROS production, and increased antioxidant enzyme activities. In addition, RSV treatment prevented EMT and fibronectin production. Renal epithelial cell apoptosis was reduced by RSV treatment through increased anti-apoptotic protein levels and reduced pro-apoptotic protein expression. Furthermore, RSV treatment increased mitochondrial membrane potential and complex III activity to attenuate the mitochondrial dysfunction and metabolic stress (Table 2). 

### 2.3. In Vitro Studies: Effects of Resveratrol on Cells of the Renal Corpuscle

Renal podocytes are cells that wrap around the capillaries of the glomerulus in the Bowman’s capsule. Functionally, podocytes, together with renal endothelial cells, form the filtration barrier and interact with mesangial cells to regulate glomeruli function [9]. In a study by Yang et al. (2013), mouse podocytes treated with TGF-β1 to induce transdifferentiation followed with RSV treatment resulted in significantly reduced albumin permeability across the podocyte monolayer, indicating reduced podocyte death and increased percentage of E-cadherin expressing cells [86]. Additionally, adhesion molecules P-cadherin, zonula occludens-1 (ZO-1), and kin of IRRE-like protein 1 (NEPH1) protein levels were significantly increased, while α-SMA protein levels were decreased with RSV treatment, indicating preserved podocyte function (Table 3) [86].

Treatment of podocytes with RSV resulted in attenuation of the high glucose-induced mitochondrial stress, decreased mROS production and increased membrane potential, all involved in diabetic nephropathy development [87]. In addition, RSV treatment increased respiratory chain complex I and III activities, while release of pro-apoptotic proteins (cytochrome C and diablo) from the mitochondria was reduced, suggesting improved mitochondrial functioning and reduced podocyte damage. Additionally, SIRT1, PGC-1α, nuclear respiratory factor 1 (NRF-1), and TFAM mRNA and protein levels were increased with RSV treatment (Table 3) [87].

Overall, these studies suggest that treatment of cells of the renal corpuscle (podocytes) with RSV preserves membrane integrity and metabolic flux. RSV treatment reduces albumin permeability and α-SMA protein levels, suggesting preserved renal functioning. Increased mitochondria complex activities and decreased mROS production indicate increased metabolic flux and decreased oxidative stress with RSV treatment. These data show that treatment of cells of the renal corpuscle with RSV exhibit a kidney oxidative protective effect and improved function (Table 3).

### 2.4. In Vitro Studies: Effects of Resveratrol on Embryonic Kidney Cells

The development of the embryonic kidney begins with the invasion of the metanephric mesenchyme by the ureteric bud. Under a series of morphogenetic events that convert the mesenchyme to epithelium, the basis of the mature nephron is formed [88]. The human embryonic kidney (HEK) 293 cell line is commonly used in research as a model of kidney cell differentiation [89]. In a study by Rössler et al. (2015), treatment of HEK293 cells with RSV resulted in increased early growth response 1 (Egr-1) protein levels and the transcription of the Egr-1 responsive reporter gene, indicating increased activity [90]. In addition, RSV treatment increased ERK1/2 phosphorylation and Raf activation, while MAP kinase phosphatase-1 (MKP-1) activity was impaired [90]. ETS like-1 protein (Elk-1) transcriptional activity was significantly increased with RSV treatment. Importantly, inhibition of ERK or use of dominant negative Raf prevented the RSV induced increased Egr-1 levels. These data suggest that RSV induces the expression of Egr-1 by ERK and Raf activation and MKP-1 repression (Table 4) [90].

Ochratoxin A (OTA) is a nephrotoxin that results in the destruction of renal tubular epithelium resulting in progressive renal failure, effects associated with decreased antioxidant activity and increased ROS production [91]. Treatment of HEK293 cells with RSV resulted in significantly decreased intracellular ROS production; however, when co-treated with OTA, RSV was unable to mitigate the increased ROS production (Table 4) [92]. DNA damage was decreased in HEK293 cells treated with RSV alone and co-treated with OTA, suggesting improved epithelium preservation. Additionally, OTA-induced 8-oxoguanine glycosylase (OGG1) mRNA levels were significantly increased with RSV, indicating increased DNA repair. OTA-induced glutathione (GSH) levels were significantly increased in cells treated with RSV, compared to OTA treated cells [92]. Overall, these data indicate that RSV treatment protects against nephrotoxin-induced DNA damage through decreased ROS production and increased antioxidant GSH level. 

Treatment of HEK293 cells with RSV resulted in significantly decreased high glucose-induced aging marker, β-galactosidase, mRNA levels, indicating reduced aging. RSV treatment also increased high glucose-induced SIRT1 and thioredoxin (Trx) mRNA levels while Trx interacting protein (TXNIP) mRNA levels were reduced indicating improved intracellular antioxidant expression (Table 4) [93].

Overall, these studies suggest that treatment of embryonic kidney cells with RSV reduced toxin or aging-induced DNA-damage and increased DNA-repair, indicative of improved cellular activity and longevity. In addition, RSV treatment reduced OTA- and high glucose-induced oxidative stress with increased GSH enzyme activity and decreased ROS production. These data show that RSV treatment protects embryonic kidney cells from DNA damage (Table 4).

**Table 4:** Effects of resveratrol on embryonic kidney cells.

Cell	Resveratrol Concentration/Duration	Effect	Reference
HEK293 cells	20 μM; 24 h	↑Egr-1 protein ↑Egr-1 reporter mRNA ↑Ph-ERK1/2 protein ↓MKP-1 activity ↑Elk-1 transcriptional activation potential	[90]
HEK293 cells	25 μM; 24–48 h	↓OTA-induced Oxidative stress DNA damage ROS production ↑OGG1 expression ↑GSH levels	[92]
HEK293 cells	2.5, 5, and 10 μM; 12–48 h	↓High glucose-induced Aging β-galactosidase mRNA TXNIP mRNA ↑SIRT1 mRNA ↑Trx mRNA	[93]

Egr-1: early growth response 1; MKP-1: MAP kinase phosphatase-1; Elk-1: ETS transcription factor; OTA: ochratoxin A; OGG1: OTA-induced 8-oxoguanine glycosylase; GSH: glutathione; Trx: thioredoxin; TXNIP: Trx interacting protein.

### 2.5. In Vitro Studies: Effects of Resveratrol on Kidney Fibroblasts

Kidney fibroblasts are found in the interstitium, are involved in the production of ECM components, such as fibronectin and collagen, and act to maintain ECM homeostasis by producing ECM-degrading proteases. With dysfunction, fibroblasts continue to produce ECM components resulting in tubulointerstitial fibrosis and renal failure [10]. Only one study exists (by He et al. (2016)) on the effects of RSV treatment on kidney fibroblast cells [94]. Treatment of NRF-49F fibroblasts with RSV resulted in the attenuation of the high glucose-induced cell proliferation and dose-dependently reduced ROS production. Additionally, RSV treatment increased phosphorylated AMPK and acetyl-CoA carboxylase (ACC) protein levels, while NOX4, α-SMA, and fibronectin protein levels were decreased back to levels similar to control cells (Table 5) [94]. These data suggest that RSV treatment increased phosphorylated AMPK and ACC reduces oxidative stress marker NOX4 activity and results in the reduction of ROS production. 

### 2.6. In Vitro Studies: Effects of Resveratrol on Renal Cancer Cells

Renal cancer accounts for more than 140,000 deaths/year, ranking as the 13th most common cause of cancer death worldwide [30,31]. Renal cancer is characterized by decreased kidney filtration, anemia, and increased blood pressure, resulting in impaired functioning and complete kidney failure [33]. Increased expression of vascular endothelial growth factor (VEGF) is associated with poor prognoses and increased metastasis [95]. Treatment of human renal cancer cells (786-0) with RSV resulted in reduced cell growth that was associated with reduced VEGF mRNA and protein levels (Table 6) [95]. Signal transducers and activators of transcription (STAT) proteins are upregulated in various malignancies, including renal cancer. Treatment of Caki-1 and 786-0 renal cancer cells with RSV promoted cell apoptosis and reduced cell survival as seen by the reduced colony formation [96]. RSV inhibited phospho-STAT3 (tyrosine 705 and serine 727), phospho-STAT5 (tyrosine 684 and tyrosine 699), and nuclear STAT3 and STAT5 protein levels, while protein tyrosine phosphatase (protein tyrosine phosphatase (PTP)ε and Src homology- 2 domain containing phosphatase (SHP-2)) mRNA and protein levels were increased [96]. Additionally, the protein levels of phosphorylated upstream kinases (Janus kinase (JAK)1, JAK2, and Src) were significantly inhibited by RSV. Bcl-2, bcl-xL, survivin, inhibitor of apoptosis (IAP)-1, and IAP-2 protein levels were reduced, while caspase-3 protein level and poly (ADP-ribose) polymerase (PARP) cleavage were increased by RSV treatment in both renal cancer cell lines (Table 6) [96].

Treatment of ACHN and A498 renal carcinoma cells with RSV resulted in significantly impaired cell growth, cell-to-cell contact, and migration (Table 6) [97]. RSV treatment inhibited the formation of filopodia, which are actin-rich microspikes that project out of the cell cytoplasm and are involved in migration. Additionally, RSV treatment reduced EMT markers (N-cadherin and vimentin), transcriptional repressor (Snail), tumor metastasis markers (MMP-2 and MMP-9), phosphorylated Akt, and ERK1/2 protein levels, while cell invasion suppressor marker (E-cadherin and tissue inhibitors of metalloproteinase 1 (TIMP-1)) protein levels were increased [97].

Overall, these studies suggest that treatment of renal carcinoma cells with RSV resulted in reduced cell proliferation, survival, and migration. RSV treatment promoted cell apoptosis and pro-apoptotic protein expression. These limited studies indicate protective effects of RSV against renal cancer (Table 6). 

### 2.7. In Vivo Animal Studies: Effects of Resveratrol on Diabetic Nephropathy

Diabetic nephropathy is a major complication of T2DM, that results in glomeruli damage and an inability to correctly filter the blood [29]. Diabetic *db/db* mice were treated with RSV (0.3% diet) for 8 weeks, resulting in decreased urinary albumin excretion that indicated increased kidney function [98]. RSV treatment also attenuated renal pathological changes with decreased mesangial cell expansion, fibronectin accumulation, and macrophage infiltration. Serum glucose, insulin, triglyceride, and free-fatty acid (FFA) levels were also decreased with RSV treatment [98]. RSV treatment also exerted antioxidant properties, through increased O^2−^ scavenging and Mn-SOD activity and decreased urinary 8-OHdG excretion. Mitochondrial biogenesis (PGC-1α, NRF-1 and cytochrome C oxidoreductase) mRNA levels, and mitochondrial DNA content were also reduced with RSV treatment, suggesting normalization of oxidative stress [98]. Surprisingly, AMPK and SIRT1 levels were unchanged in diabetic *db/db* mice treated with RSV, suggesting that RSV mediates mitochondrial biogenesis independent of AMPK and SIRT1 activation (Table 7) [98]. 

Chronic RSV administration (5 mg/kg/day; 16 weeks) in streptozotocin (STZ)-induced diabetic rats resulted in attenuation of the diabetic phenotype with reduced serum glucose levels and maintained body weight [99]. RSV treatment significantly increased serum SOD activity, while thiobarbituric acid reactive substances (TBARS) level and GSSG/GSH ratio were decreased. Pro-inflammatory cytokine TNF-α and IL-6 plasma concentrations were significantly reduced with RSV treatment, indicating anti-inflammatory effects. In addition, RSV treatment reduced NF-κB activity in polymorphonuclear cells. Apoptosis rate was significantly reduced in polymorphonuclear cells, kidney, liver, heart and sciatic nerve with RSV treatment (Table 7) [99].

In a study by Jiang et al. (2013), administration of RSV (20 mg/kg/day) for 8 weeks to STZ-induced diabetic Wistar rats resulted in significantly reduced plasma glucose, creatinine, and urinary protein excretion [100]. RSV treatment also attenuated the diabetic-induced mesangial cell hyperplasia and mesangial matrix expansion. Glutathione S-transferase Mu (GSTM) protein levels in diabetic rats were significantly reduced with RSV treatment (Table 7) [100].

In a study by Kim et al. (2013), treatment of *db/db* mice with RSV (20 mg/kg/day) for 12 weeks resulted in reduced lipotoxicity-related apoptosis of renal cells. RSV treatment significantly reduced kidney non-esterified fatty acid (NEFA) and triacylglycerol content and reduced albuminuria production [101]. Mesangial area, TGF-β1, type IV collagen and F4/80 positive cells were significantly reduced with RSV treatment, indicating reduced renal diabetic phenotypic changes. RSV treatment increased phosphorylated AMPK, ERR-1a, and SIRT1 protein levels, while PPARα-estrogen-related receptor-1α-sterol regulatory element-binding protein 1 (SREBP1) levels were decreased [101]. Phosphorylated Akt (S473), phosphorylated FOX03α, and PI3K protein levels and PI3K activity were decreased with RSV treatment [93]. RSV treatment increased Bcl-2 and decreased Bax and cleaved caspase-3 protein levels. RSV treatment decreased renal and urinary 8-OHdG concentrations, indicating decreased renal oxidative stress DNA damage (Table 7) [101].

Diabetes was induced in FVB mice with five intraperitoneal injections of STZ followed by 12 weeks of RSV (10 mg/kg/day) administration [70]. RSV administration prevented glomerular enlargement and extracellular matrix protein accumulation and reduced urinary albumin levels, suggesting preserved kidney functioning (Table 7). Kidney phosphorylated Akt, PAI-1, and intercellular adhesion molecule 1 (ICAM-1) protein levels were reduced with RSV treatment. Additionally, glomeruli PCNA mRNA levels, a marker of cell proliferation, were decreased with RSV treatment, suggesting that RSV protects against diabetes-induced kidney inflammation and cell proliferation [70]. 

In a study by Ji et al. (2014), treatment of STZ-induced diabetic rats with RSV for 12 weeks resulted in significant attenuation of renal pathological changes with reduced renal glomeruli area, reduced mesangial cell expansion and reduced the thickness of the glomerular basement membrane in the kidney [69]. Additionally, RSV treatment increased AdipoR1 expression, and reduced MDA generation, fibronectin, and collagen IV expression in the glomeruli (Table 7) [69].

Administration of RSV (10 mg/kg/day) for 30 days to STZ-induced diabetic nephropathic Wistar rats resulted in the attenuation of the diabetic phenotype with decreased serum glucose, urea nitrogen, and urine MDA levels [102]. Kidney tissue antioxidant SOD and catalase activities were increased with RSV treatment. STZ-induced histopathological alterations, epithelial desquamation, swelling, intracytoplasmic vacuolization, brush border loss, peritubular infiltration and glomerulus sclerotic presence were significantly reduced with RSV treatment, suggesting preserved kidney structure and functioning. In addition, RSV treatment significantly decreased the number of transforming growth factor-β1 positive cells in the kidney (Table 7) [102].

Administration of RSV (40 mg/kg/day) to *db/db* diabetic mice for 12 weeks resulted in renal protection through improved body weight and decreased albuminuria, serum urea nitrogen, and creatinine levels [103]. RSV treatment also significantly reduced glomerulosclerosis and tubulointerstitial fibrosis. Kidney SOD Cu/Zn, Mn-SOD, and catalase protein levels were increased in RSV-treated animals, while renal MDA protein levels were decreased, indicating protective properties of RSV against renal oxidative stress. RSV treatment attenuated the diabetes-induced increase in α-SMA and decreased E-cadherin kidney protein levels, indicating preserved structure [103]. Additionally, RSV treatment decreased kidney tissue TGF-β, pSmad3, phosphorylated Akt, and ERK protein levels and IGF-1 receptor (IGF-1R) expression, while 3-hydroxy-3-methylglutaryl reductase degradation (HRD1) expression was increased (Table 7) [103].

Administration of RSV (20 mg/kg/day) to diabetic *db/db* mice for 12 weeks resulted in reduced glomerular matrix expansion and albuminuria. RSV treatment increased serum adiponectin levels and renal cortex AdipoR1 and AdipoR2 protein levels, suggesting regulation of lipid and glucose homeostasis [104]. Pro-apoptotic Bax protein level was decreased, while the anti-apoptotic Bcl-2 protein level was increased with RSV treatment, suggesting attenuation of diabetes-induced kidney cell apoptosis. RSV treatment also significantly diminished renal and urinary 8-OHdG and urinary 8-isoprostane levels, indicating reduced renal oxidative stress and lipid peroxidation [104]. Renal cortical phosphorylated AMPK (T172), PPARα, total FoxO1, total FoxO3a, and SIRT1 protein levels were significantly increased with RSV treatment. Additionally, PGC-1α, ERR-1α, and phosphorylated ACC protein levels were increased, while SREBP-1c protein levels, NEFA, and triglyceride content in the renal cortex was reduced with RSV treatment (Table 7) [104].

In a study by He et al. (2016), RSV (40 mg/kg/day) administration to *db/db* mice for 12 weeks resulted in significantly reduced mesangial area and collagen deposition in the renal interstitium and urine albumin levels [94]. Additionally, RSV treatment significantly reduced fibroblast-specific protein 1 (FSP-1), α-SMA, and fibronectin protein levels, indicating decreased proliferation and activation of renal fibroblasts. The NOX4 protein level was significantly reduced in renal tissues and renal interstitium, while phosphorylated AMPK and ACC protein levels were increased with RSV treatment (Table 7) [94].

RSV (5 mg/kg/day) administration to STZ-induced diabetic Sprague–Dawley rats for 4 months resulted in improved renal function and reduced blood glucose, lipid, hemoglobin A1c (HbA1c), urea nitrogen, and creatinine levels [105]. RSV treatment restored renal functioning with decreased urinary albumin level. In addition, RSV treatment significantly reduced serum pro-inflammatory cytokine (TNF-α, IL-6, IL-1β, and IL-10) and renal 8-OHdG levels, indicating reduced inflammation and improved mitochondrial function, respectively. RSV treatment promoted Sirt1 mRNA and protein levels and improved autophagy activity with increased Atg5 and Atg7 mRNA in the diabetic kidney (Table 7) [105].

In a study by Wang et al. (2017), treatment of STZ-induced diabetic Wistar rats with RSV (30 mg/kg/day) for 16 weeks resulted in improved renal function and histopathology with reduced kidney and body weight, and increased creatinine clearance rate (Table 7) [83]. RSV treatment also reduced the high glucose-induced oxidative stress through increased SOD and SIRT1 activity and CAT protein levels, while MDA kidney content was reduced. The increased SIRT1 activity correlated with reduced acetylated-FOXO3a in the kidneys of diabetic rats treated with RSV [83]. 

Treatment of STZ-induced diabetic Sprague–Dawley rats with RSV (20 mg/kg/day) for 4 weeks resulted in reduced fibronectin accumulation and preserved kidney function [71]. Serum glucose and creatinine level, urinary albumin levels and kidney weight were also decreased with RSV treatment, attenuating the diabetic phenotype. RSV treatment reduced glomerular thickening, interstitial fibrosis, epithelial cellular vacuolar degeneration, hyaline casts and arteriolopathy in diabetic kidneys. Additionally, p38, phosphorylated p38, TGF-β1, and fibronectin protein levels were reduced in the distal tubules, collecting ducts, proximal tubes and glomeruli with RSV treatment, indicating improved kidney function (Table 7) [71].

RSV (5 mg/kg/day) administration for 45 days in STZ-induced diabetic Wistar rats resulted in reduced renal hypertrophy, mesangial expansion, reduced fibrosis, and glomerulonephritis [106]. Advanced glycation end production (AGE) accumulation in the mesangium, vascular endothelium, and proximal convoluted tubules of the diabetic kidney was significantly reduced with RSV treatment. In addition, oxidative stress marker (8-oxo-dG) levels in nuclei and cytoplasm of renal cells and levels of the lipid peroxidation marker (4-hydroxynonenal (HNE)) in glomeruli, convoluted tubules, loops of Henle, and collecting ducts were reduced with RSV treatment. RSV treatment significantly reduced caspase-3 and cleaved caspase-3 protein levels, indicating reduced apoptosis (Table 7) [106]. Therefore, RSV alleviates the diabetes-induced glycation, oxidative damage, and apoptosis to inhibit diabetic nephropathy progression. 

In a study by Guo et al. (2018), administration of RSV (10 mg/kg/day) for 8 weeks in C57BL/KsJ *db*/+ mice resulted in reduced fasting serum glucose, insulin, and pro-inflammatory cytokine (IL-1β, IL-17, IL-10, and TNF-α) levels, while IL-6 and VEGF serum levels were increased [107]. Additionally, RSV treatment reduced the number of apoptotic renal cells and inhibited mRNA levels of apoptosis-associated genes (Apaf-1, caspase-3, caspase-8, and caspase-9). Oxidative stress total thiol levels were decreased in renal cells treated with RSV, while antioxidant GSH levels were increased [107]. Renal phosphorylated and total AMPK protein levels were decreased by RSV treatment (Table 7) [107]. 

Treatment of STZ-induced CD-1 diabetic mice with RSV (30 mg/kg/day) for 12 weeks resulted in significantly reduced kidney injury and cellular apoptosis. RSV treatment decreased serum glucose, cholesterol and urea nitrogen levels [87]. In addition, RSV treatment reduced diabetic kidney injury with reduced glomerular thickening and mesangial area, while the podocyte mitochondria number was increased. RSV treatment also provided antioxidant effects with decreased MDA content and increased Mn-SOD activity in the renal cortex [87]. Nephrin, SIRT1, PGC-1α, NRF1, and TFAM protein levels were decreased, while cleaved caspase-3 protein levels were increased in the renal cortex with RSV treatment. SIRT1 and NRF1 expression was also increased within the nucleus of glomerular podocytes, mesangial cells, and renal tubular epithelial cells. In the glomeruli and tubulointerstitium the number of apoptotic cells was significantly reduced with RSV treatment, indicating anti-apoptotic properties associated with RSV (Table 7) [87]. 

Overall, these studies suggest that treatment of animals suffering from diabetic nephropathy with RSV attenuates hyperglycemia, hyperlipidemia and improves kidney structural integrity and kidney function. RSV administration decreased urinary albumin and serum creatinine levels, indicating improved kidney functioning. In addition, renal oxidative stress, inflammatory cell infiltration, cytokine production, and MDA content were reduced with RSV administration, while antioxidant enzyme activity and SIRT1 expression were increased. These data show that RSV treatment has protective effects against diabetic nephropathy (Table 7).

### 2.8. In Vivo Animal Studies: Effects of Resveratrol on Renal Fibrosis

Treatment of ethylene glycol (EG)-treated Sprague–Dawley rats, a rat model of kidney stones, with RSV (10 mg/kg/day) for 21 days resulted in decreased urinary crystals and serum MDA levels [75]. Additionally, RSV treatment increased kidney antioxidant enzymes (glutathione peroxidase, catalase, and SOD) protein levels. Hyaluronan and osteopontin kidney proteins levels were decreased with RSV treatment, indicating reduced renal cell injury caused by EG (Table 8) [75].

In a study by Zhang et al. (2014), treatment of arsenic-trioxide (As_2_O_3_)-induced renal fibrotic Wistar rats with RSV (8 mg/kg/alternate day) for 8 days resulted in decreased oxidative stress with decreased MDA and ROS production and increased SOD and GPx levels in the kidney (Table 8) [108]. The percentage of necrotic renal tubular epithelial cells was significantly reduced, while selenium content in the kidneys was increased with RSV treatment, indicating improved defense against oxidative damage. Biochemical alterations in BUN and creatinine levels were significantly reduced with RSV administration. Kidney morphological alterations induced by As_2_O_3_ were also reduced with RSV treatment. In addition, serum urea nitrogen and creatinine levels were significantly reduced with RSV treatment, indicating improved renal functioning [108]. 

RSV administration (20 mg/kg/day) for 14 days in unilateral ureteral obstructed (UUO) C57BL/6J mice attenuated renal injury with reduced extracellular matrix protein deposition, and tubulointerstitium damage (Table 8) [109]. Additionally, RSV treatment reduced ROS production and 8-OHdG levels, while SOD levels were increased, indicating renal protection from ROS damage. Renal cortical TNF-α, ICAM-1, and TGF-β mRNA levels and fibronectin and acetyl-Smad3 protein levels were reduced by RSV treatment [109].

Treatment of UUO-rats with RSV (20 mg/kg/day) for 14 days resulted in significantly reduced renal interstitium damage with decreased inflammatory cell infiltration and tubular dilation and atrophy [77]. Collagen deposition in the renal interstitium was also reduced with RSV treatment. In addition, serum creatinine levels, kidney tissue TGF-β1 levels, mesenchymal markers, α-SMA, and type III collagen mRNA and protein levels were decreased, while the mRNA and protein levels of the epithelial marker E-cadherin were increased with RSV treatment (Table 8) [77].

RSV administration (20 mg/kg/day) for 6 weeks in ischemia-reperfusion (I/R) mice and UUO mice resulted in decreased serum creatinine and BUN levels [79]. In addition, α-SMA and COL1A1 kidney protein levels were increased with RSV treatment (Table 8) [79].

Sprague–Dawley rats were induced septic by cecal ligation and puncture (CLP). A single injection of RSV (50 mg/kg) in these septic rats resulted in increased renal epithelial cell SIRT1 and SIRT3 activity and protein levels [110]. SOD2 is a target for deacetylation by SIRT1/3, and RSV treatment decreased acetylated-SOD2 levels; however, SOD2 protein content was increased. In addition, other antioxidants, GSH and CAT, content and activity were increased with RSV treatment [103]. Mitochondrial content and transmembrane potential were significantly increased with RSV injection, while mitochondrial permeability transition pore (mPTP) opening was reduced, indicating reduced renal damage. The number of terminal deoxynucleotidyl transferase dUTP nick end label (TUNEL) positive cells, as an indicator of apoptosis, was reduced in the kidney of rats injected with RSV. This was accompanied with increased kidney functioning, through decreased serum creatinine and urea nitrogen levels [110]. 

In a study by Wu et al. (2016), RSV (200 mg/kg/day) administration for 5 weeks in male cystic (Cy/+) rats resulted in decreased polycystic kidney disease progression [81]. RSV treatment significantly reduced cyst wall thickness/complexity and decreased BUN and creatinine serum levels, indicating improved kidney function. Macrophage infiltration and pro-inflammatory factor MCP-1, TNF-α, and complement factor B (CFB) protein levels, were reduced with RSV treatment in Cy/+ kidneys. Additionally, phosphorylated NF-κB (p65 and p50) and S6K protein levels were reduced in the kidney with RSV treatment (Table 8) [81].

Acute kidney injury and excessive systemic inflammatory response are common complications of sepsis. Sprague–Dawley rats with sepsis induced through cecal ligation and puncture were treated with a single intraperitoneal injection of 3 and 10 mg/kg RSV for 70 h which resulted in improved survival [111]. RSV administration reduced serum BUN, creatinine, and nitrogen levels and decreased renal injury index [111]. Serum levels of cystatin C, a biomarker of kidney dysfunction, were also significantly reduced with RSV treatment, indicating improved kidney function. In addition, pro-inflammatory cytokine (TNF-α, IL-1β, IL-6) and kidney injury molecule-1 (KIM-1) serum levels were reduced with RSV treatment (Table 8) [111]. 

Treatment of cadmium-induced Kumming mice with RSV (10 mg/kg/day) for 1 week resulted in reduced BUN and serum creatinine levels [84]. Additionally, RSV treatment decreased caspase-3 activity and Bax protein levels, indicating reduced kidney cell apoptosis. RSV treatment markedly reduced phosphorylated ERK1/2 protein level in cadmium-treated kidneys (Table 8) [84].

A single intraperitoneal injection of RSV (30 mg/kg) in male AKI rats, a model of polymicrobial sepsis, resulted in increased renal function and survival with reduced serum creatinine and urea nitrogen levels and reduced tubular epithelial cell injury [82]. Renal tubular injury (heme oxygenase-1 (HO-1), KIM-1, and neutrophil gelatinase-associated lipocalin (NGAL)) mRNA markers were significantly reduced with RSV treatment. In addition, serum and kidney tissue pro-inflammatory cytokines TNF-α, IL-1β, and IL-6 mRNA and supernatant content were reduced with RSV treatment. The percentage of positive renal nuclear p65 expressing cells and kidney protein levels of phospho-IRE1 were significantly reduced with RSV treatment (Table 8) [82]. 

Treatment of 5/6 nephrectomized Sprague-Dawley rats, a rat model for CKD, with RSV (20 mg/kg/day) for 4 weeks resulted in attenuated glomerular injury with decreased mesangial cell proliferation and glomeruli matrix expansion [72]. Kidney TGF-β protein levels were significantly decreased with RSV treatment. Renal cortex mitochondrial electron transport chain proteins, ATP synthase B and cytochrome c oxidase subunit I (COX I), protein levels were increased with RSV treatment, while ROS production was decreased [72]. In addition, RSV treatment increased kidney cortex ATP production and mitochondrial complex I and III enzymatic activity. Mitochondrial fusion was favored with RSV treatment, due to increased optic atrophy 1 (Opa1) and mitofusin 2 (Mfn2) protein levels and decreased fission dynamin-related protein 1 (Dnp1) protein levels (Table 8) [72]. 

In a study by Liu et al. (2018), treatment of renal injured (UUO) C57BL/6 mice with RSV (25 mg/kg/day) for 2 weeks resulted in reduced serum creatinine levels and reduced kidney α-SMA, Snail and fibronectin protein levels (Table 8) [85]. Tubular lesion score and interstitial collagen deposition were significantly reduced in the kidneys of animals treated with RSV. This was associated with SIRT1 upregulation and reduced phosphorylated Smad3, indicating partial reduction of renal fibrosis by low (25 mg/kg/day) RSV treatment. In contrast, a high dose of RSV (100 mg/kg/day) resulted in aggravated renal fibrosis that was associated with increased kidney α-SMA and TFAM expression [85]. These data suggest that RSV at high doses may exert pro-fibrotic effects and further studies are required to investigate the potential RSV concentrations that exert nephroprotective effects and those that may have potential nephrotoxicity. 

Overall, these studies suggest that administration of RSV to animal models of renal fibrosis reduced extracellular matrix protein deposition, reduced tubulointerstitium damage, and mesangial cell proliferation. RSV reduced serum creatinine levels and kidney oxidative stress, while kidney antioxidant enzymes (SOD, CAT, GPx, and GSH) were increased. In addition, RSV treatment improved mitochondrial biogenesis, mitochondrial complex I and III activities, and electron transport protein expression, while mPTP opening and fission protein expression were reduced. RSV treatment also exerted anti-inflammatory effects, by reducing mRNA and protein expression of pro-inflammatory signaling molecules and cytokines. These data demonstrate that RSV treatment exerts protective effects against renal fibrosis (Table 8). 

## 3. Effects of Resveratrol on Human Kidneys

Only two clinical studies exist measuring the effects of RSV in humans with kidney disease. In a randomized, double-blinded pilot study by Saldanha et al. (2016), administration of RSV (500 mg/day) for 4 weeks to non-dialyzed chronic kidney disease (CKD) patients (GFR between 15 and 60 mL/min/m^2^) resulted in no significant effects. Antioxidant and anti-inflammatory marker levels were the same in RSV and placebo supplemented participants (Table 9) [112]. It should be emphasized that administration of RSV (500 mg/day) for 4 weeks had low toxicity. 

In another randomized, double-blinded study by Lin et al., low-dose (150 mg/day) or high-dose (450 mg/day) of RSV intake for 12 weeks by peritoneal dialysis (PD) patients resulted in significant improvements in mean net ultrafiltration (UF) volume and rate (Table 9) [113]. In addition, angiogenesis markers, VEGF, fetal liver kinase-1 (Flk-1), and angiopoietin (Ang)-2 levels in peritoneal dialysate effluent (PDE) were significantly reduced in the high-dose RSV group. The levels of angiopoietin receptor (Tie-2) and thrombospondin-1 (Tsp-1) in the PDE were increased with RSV treatment [113]. These data suggest that RSV treatment has angiogenesis-ameliorating effects in PD patients and improves ultrafiltration kidney function. It should be mentioned that in the study by Saldanha et al. [112] administration of 500 mg RSV/day for 4 weeks resulted in no significant effects, while in the study by Lin et al. [113] administration of 450 mg RSV/day for 12 weeks resulted in significant improvements and health benefits, suggesting that longer duration of administration of a specific dose of RSV (450 or 500 mg/day) may be required to see/elicit beneficial effects. 

Other clinical studies exist showing beneficial effects of RSV administration in cardiovascular disease, diabetes mellitus and cancer, however, the effect of RSV supplementation in kidney disease patients has not been extensively studied [114,115]. In a randomized, double-blinded study by Brasnyo et al. (2011), oral administration of RSV (10 mg/day) in type 2 diabetic (T2DM) individuals (following the WHO diagnostic guidelines), significantly increased insulin sensitivity and reduced serum glucose and cholesterol levels [116]. In addition, RSV treatment significantly reduced serum creatinine levels and maintained GFR, suggesting improved kidney function [116]. In a similar randomized, open-label, controlled study, administration of RSV (250 mg/day) for 4 months in T2DM patients (3 year duration of T2DM and minimum 6 months oral hypoglycemic treatment) resulted in significantly improved lipid profile, with reduced total cholesterol and triglyceride levels [117]. Serum creatinine, urea nitrogen levels, and total protein excretion were reduced with RSV treatment, suggesting improved kidney function [117]. These studies [116,117] show that treatment of individuals with T2DM and impaired kidney function with RSV resulted in improved glucose, insulin, and lipid homeostasis and better kidney function.

Although there are numerous studies measuring the effects of RSV in diabetes, the studies mentioned above were performed in individuals with established CKD and diabetic nephropathy and show a kidney-protective effect of RSV administration. These data highlight the importance of future clinical trials required to investigate the exact effects of RSV in individuals with kidney disease. 

**Table 9:** Effects of resveratrol on human kidneys.

Patients	Resveratrol Concentration/Duration	Effect	Reference
Nondialyzed CKD patients	500 mg/day; 4 weeks	No significant effects	[112]
PD patients	150 and 450 mg/day; 12 weeks	↓UF volume and rate ↓PDE VEGF, Flk-1 and Ang-2 ↑PDE Tie-2 and Tsp-1	[113]
T2DM patients	10 mg/day; 4 weeks	↑Kidney filtration ↑Insulin sensitivity ↓Glucose levels ↓Lipid levels ↓Serum creatinine	[116]
T2DM patients	250 mg/day; 4 months	↑Kidney function ↓Cholesterol levels ↓Triglyceride levels ↓Serum creatinine ↓Total protein excretion ↓Urea nitrogen levels	[117]

CKD: chronic kidney disease; PD: peritoneal dialysis; UF: ultrafiltration; PDE: peritoneal dialysate effluent; Flk-1: fetal liver kinase 1; Ang: angiopoietin; Tie-2: angiopoietin receptor; Tsp-1: thrombospondin-1; T2DM: type 2 diabetes mellitus.

## 4. Effects of RSV at the Cellular/Molecular Level

**Figure 2:**
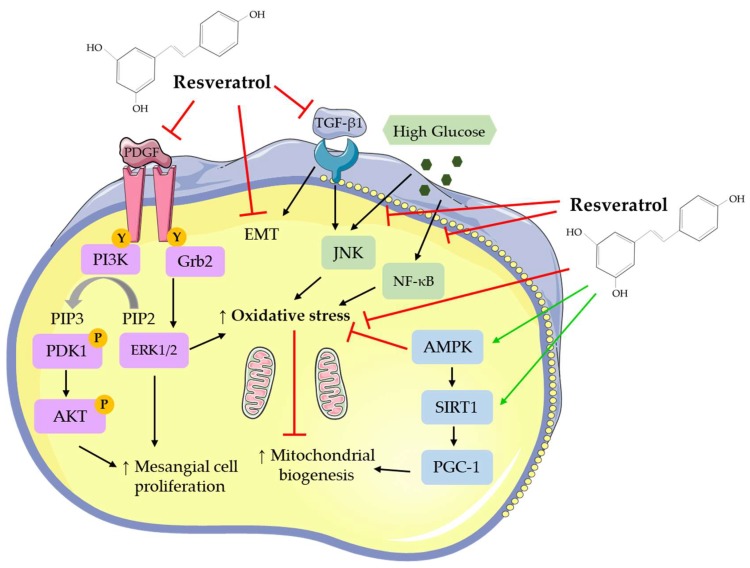
Effects of resveratrol on cellular signaling molecules. The figure was created based on the data of the studies [66,76,80,81,85,91,95,101,102]. AKT: protein kinase B; PDK: pyruvate dehydrogenase kinase; PIP3: phosphatidylinositol-3,4,5-triphosphate; PIP2: phosphatidylinositol 4,5-bisphosphate; ERK: extracellular signal-regulated kinase; PI3K: phosphoinositide 3-kinase; Grb: growth factor receptor-bound protein; PDGF: Platelet-derived growth factor; EMT: extracellular matrix transition; JNK: c-Jun N-terminal kinase; AMPK: AMP-activated protein kinase; SIRT: sirtuin; PGC: Peroxisome proliferator-activated receptor gamma coactivator; NF-κB: nuclear factor kappa-light-chain-enhancer of activated B cells; TGF-β: transforming growth factor beta.

Resveratrol has been found to affect a number of different signaling molecules in kidney cells (Figure 2). RSV inhibited the PDGF [66] and TGF-β1 response in mesangial [71,72,102] and epithelial [75,79,85] cells. It decreased oxidative stress [75,77,81,84,92,101,102], as shown by decreased ROS and MDA levels and increased antioxidant enzyme activity and improved mitochondrial biogenesis [67,72,85] (Figure 2). Activation of the energy sensor AMPK [73,76,94,101] and increased SIRT1 [72,74,79,87,101] and PGC-1 [84,87] levels were seen with RSV treatment. The deleterious effects of high glucose on kidney cells were diminished with RSV treatment [67,68,78,83,87,93,94] (Figure 2). 

## 5. Conclusions and Future Directions

Overall, all available in vitro and in vivo animal and human studies examining the effects of RSV in kidney disease indicate that it can reduce fibrosis, mesangial expansion, oxidative stress, and inflammatory cytokine levels, while improving kidney structure and function. Treatment of renal mesangial, epithelial, and corpuscle cells with RSV resulted in reduced structural changes and ROS production, while antioxidant and mitochondrial activities were improved. In addition, RSV treatment reduced fibroblast proliferation and activation to improve kidney structural maintenance. Renal cancer cells treated with RSV had reduced cell growth, cell-to-cell contact, and migration, and increased apoptosis.

In in vivo animal models of diabetic nephropathy treatment with RSV showed improved glucose homeostasis, reduced inflammation and increased antioxidant activity and kidney function. Animals with renal fibrosis administered RSV had reduced structural changes and inflammatory cell infiltration, cytokine expression, and decreased tubulointerstitium damage and oxidative stress. 

The limited human studies indicate a protective effect of RSV administration on chronic kidney disease with increased kidney filtration rates and volume. The health benefits of RSV are widespread, and the low toxicity of the molecule makes it a prime candidate for medicinal use against kidney disease. However, more research and clinical studies are required to fully understand the effects of RSV on kidney disease.

Further investigation and clarification are required in the following areas: (1) dosage and bioavailability, (2) metabolism, tissue distribution, and biological effects of RSV analogs and metabolites, and (3) signaling mechanisms involved. 

Only limited number of studies exist examining RSV administration in humans. More studies should be performed to determine the optimal dosage and route of administration of RSV and analogs with higher biological activity. RSV analogs (methylated and with other novel derivatives) may have great biological activity [54]. Most in vitro studies and evidence have used RSV and not its metabolites. The potential biological activity of RSV metabolites should be considered in future investigations. 

Furthermore, future research should be conducted examining the exact signaling/cellular mechanisms affected by RSV and contributing to the attenuation of kidney disease.

## Figures and Tables

**Table 1:** Effects of resveratrol on kidney mesangial cells.

Cell	Resveratrol Concentration/Duration	Effect	Reference
Rat primary mesangial cells and LLCPK1 cells	50–75 µM; 24 h	↑NF-κB activation	[63]
Rat primary mesangial cells	10 µM; 1 h	↓Gentamicin-induced contraction	[65]
Rat mesangial cells	10 µM; 1 h	↓PDGF-induced cell proliferation ↓PDGFR Y-751 phosphorylation ↓PDGFR Y-761 phosphorylation ↓PDGF-induced PI3K, Akt, ERK1/2, c-Src activity ↑PTP1B activity	[66]
Rat primary mesangial cells	10 μM; 6 h	↓High glucose-induced ROS production Mitochondrial superoxide ↑ MnSOD activity ↑ Mitochondrial complex III activity ↑ ∆Ψm hyperpolarization ↑ SIRT1 activity	[67]
CRL-2573 and primary mesangial cells	5–10 µM; 24 h	↓ High glucose-induced Cell proliferation Fibronectin protein JNK and NF-κB activation NADPH oxidase activity ROS production	[68]
HBYZ-1 cells	20 μM; 72 h	↑High glucose-induced AdipoR1 mRNA and protein ↑FOX01 activity ↓FOX01 phosphorylation	[69]
Rat mesangial cells	25 μM; 48 h	↓High glucose-induced Cell proliferation PAI-1 protein Ph-Akt NF-κB	[70]
CRL-2573 cells	10 µM; 48 h	↓High glucose-induced p38 MAPK activation TGF-β1 expression Fibronectin	[71]
SV40 MES 13 cells	10 µM; 46 h	↓TGF-β1-induced ROS production ↑TGF-β1-induced Mitochondrial membrane potential ATP Complex I/III activity NDUFB8 and ATP β protein SIRT1 PGC-1α deacetylation	[72]

NF-κB: nuclear factor kappa light-chain-enhancer of activated B cells; PDGF: platelet-derived growth factor; PI3K: phosphoinositide 3-kinase; Akt: protein kinase B; ERK1/2: extracellular signal-regulated kinases 1/2; c-Src: proto-oncogene tyrosine-protein kinase Src; PTP1B: tyrosine-protein phosphatase non-receptor type 1B; MnSOD: manganese superoxide dismutase; ROS: reactive oxygen species; SIRT1: sirtuin 1; JNK: c-Jun N-terminal kinase; NADPH: nicotinamide adenine dinucleotide phosphate; FOX01: forkhead box 01; PAI-1: plasminogen activator inhibitor 1; MAPK: mitogen-activated protein kinase; TGF-β1: transforming growth factor-β1; ATP: adenosine triphosphate; NDUFB8: NADH:ubiquinone oxidoreductase subunit B8; PGC-1α: peroxisome proliferator-activated receptor gamma coactivator. ↓: decrease; ↑: increase.

**Table 2:** Effects of resveratrol on renal epithelial cells.

Cell	Resveratrol Concentration/Duration	Effect	Reference
Rodent glomerular epithelial cells	30 μM and 50 μM; 72 h	↓High glucose-induced de novo protein synthesis Acetylation of LKB1 Ph-eIF4E protein eIF4G, eEF2, and p70S6K protein	[73]
Mouse proximal tubular epithelial cells	100 μM; 30 min	↓Cisplatin-induced Apoptosis p53(S379) acetylation PUMA-α and caspase-3 protein Bax translocation ↑SIRT1 siRNA-acetylation ↑Bcl-xL, Bax, and Bak protein	[74]
Human renal epithelial cells	0, 40 and 80 µM; 24 h	↓Oxalate-induced Colonization Hyaluronan ROS production NADPH p22 and p47 mRNA MCP-1 and osteopontin mRNA TGFβ1, TGF-RI/II Malondialdehyde	[75]
mpkCCD_C14_ cells	25–400 µM; 30 min to 24 h	↓Sodium transport ↑GFP-AKT-PH redistribution ↑AMPKα protein	[76]
NRK-52E cells	10 and 100 μM; 24 h	↓TGF-β1-induced Cellular proliferation EMT EM synthesis Shh and Gli1 mRNA	[77]
HK-2 cells	5–20 µM; 4 h	↓High glucose-induced EMT ROS levels NOX1 and NOX4 protein ERK1/2 activation	[78]
HK-2 cells	20 μM; 48 h	↓EMT ↓β-catenin nuclear translocation ↑E-cadherin and SIRT1 mRNA and protein ↓MMP7, α-SMA, and COLIA1 mRNA and protein	[79]
HK-2 cells	12.5 µM; 48 h	↓Ioxitalamate-induced Cytotoxicity Cytosolic DNA fragmentation 8-OHdG formation ROS production ↑Bcl-2 and survivin protein ↑ Caspase 3 activity	[80]
OX161 and UCL93 human renal epithelial cells; MDCK canine renal epithelial cells	2–50 µM; 48 h	↓Cyst number ↓MCP-1 protein and activity ↓TNF-α protein and activity ↓CFB protein and activity ↑SOD2 protein	[81]
HK-2 cells	20 µM; 12 h	↑Cell viability ↓Ph-NFκB protein ↓TNF-α, IL-1β, and IL-6 mRNA and protein ↓IRE1 activation	[82]
HK-2 cells	25 µM; 72 h	↓High glucose-induced oxidative stress ↓MDA and ROS activity ↑CAT and SIRT1 protein ↑SIRT1 activity ↓Acetyl-FOXO3a protein	[83]
TCMK-1 cells	25 µM; 72 h	↓Cadmium-induced apoptosis ↓mROS production ↑mSIRT3 protein and activity ↑PGC-1α and SOD2 mRNA	[84]
HK-2 cells	5–20, 40 µM; 72 h	5–20 µM RSV: ↓TGF-β-induced EMT ↓Cytotoxicity ↑SIRT1 and E-cadherin protein ↓α-SMA and fibronectin protein ↓Ph-Smad3 ↓SIRT1-Smad3/4 40 µM RSV: ↑Cytotoxicity ↑mtROS release ↑Bax, fibronectin, and α-SMA protein ↓Bcl-2 protein ↓ATP production ↓PGC-1α and TFAM protein	[85]

LKB1: liver kinase B1; eIF: eukaryotic translation initiation factor; eEF2: eukaryotic translation elongation factor 2; p70S6K: ribosomal protein S6 kinase beta-1; AMPKα: AMP-activated protein kinase alpha; PUMA: pro-apoptotic p53 upregulated modulator of apoptosis; siRNA: small interfering RNA; GFP: green fluorescent protein; EMT: epithelial-to-mesenchymal transition; MDA: malondialdehyde; TFAM: mitochondrial transcription factor A; Bcl-xL: B-cell lymphoma-extra-large; Bax: Bcl-2-associated X; Bak protein: BCL2-antagonist/killer protein; α-SMA: α-smooth muscle actin; COLIA1: collagen type I alpha 1; NOX: NADPH oxidase; MMP: matrix metalloproteinase; MCP-1: monocyte chemoattractant protein 1; CAT: catalase; CFB: complement factor B; mROS: mitochondrial ROS; IL: interleukin; TFAM: mitochondrial transcription factor A.

**Table 3:** Effects of resveratrol on cells of the renal corpuscle.

Cell	Resveratrol Concentration/Duration	Effect	Reference
Mouse podocytes	2–5 µM; 30 min	↓Albumin permeability ↓Podocyte death ↑E-cadherin expression ↑P-cadherin, ZO-1, and NEPH1 protein ↓α-SMA protein	[86]
Immortalized podocytes	10 μM; 48 h	↓High glucose-induced Mitochondrial stress mROS production Cyto C and diablo release ↑Complexes I and III activities ↑Mitochondrial membrane potential ↑SIRT1, PGC-1α, NRF1, TFAM mRNA and protein	[87]

ZO-1: zonula occludens-1; NEPH1: kin of IRRE-like protein 1; NRF1: nuclear respiratory factor 1.

**Table 5:** Effects of resveratrol on kidney fibroblasts.

Cell	Resveratrol Concentration/Duration	Effect	Reference
NRF-49F cells	5, 10, and 20 µM; 1 h	↓High glucose-induced Cell proliferation Fibronectin protein α-SMA protein ROS production NOX4 protein ↑High glucose-induced Ph-AMPK Ph-ACC	[94]

ACC: acetyl-CoA carboxylase.

**Table 6:** Effects of resveratrol on renal cancer cells.

Cell	Resveratrol Concentration/Duration	Effect	Reference
786-0 cells	0, 10, 20 and 40 µM; 24, 48 and 72 h	↓Cell growth ↓VEGF mRNA and protein	[95]
Caki-1 and 786-0 cells	0, 10, 30 and 50 µM; 6 h	↑Apoptosis ↓Survival ↓Migration ↓STAT3 and STAT5 activation ↑PTPε and SHP-2 protein ↓JAK1, JAK2, and c-Src protein ↓Bcl-2, bcl-xL, survivin, IAP-1, and IAP-2 protein ↑Caspase-3 protein	[96]
ACHN and A498 cells	50 μM; 12 h	↓Cell growth ↓Cell-to-cell contact ↓Migration ↓Filopodia formation ↓N-cadherin, vimentin, snail, MMP-2, MMP-9, ph-Akt and ph-ERK1/2 protein ↑E-cadherin and TIMP-1 protein	[97]

VEGF: vascular endothelial growth factor; STAT: Signal transducers and activators of transcription; PTP: protein tyrosine phosphatase; SHP-2: Src homology- 2 domain containing phosphatase; JAK: Janus kinase; IAP: inhibitor of apoptosis; TIMP: tissue inhibitors of metalloproteinase 1.

**Table 7:** Effects of resveratrol on diabetic nephropathy (animal studies).

Animal	Resveratrol Concentration/Duration	Serum Effects	Other Effects	Reference
db/db mice	0.3% diet; 8 weeks	↓Glucose levels ↓Insulin levels ↓Triglyceride levels ↓FFA levels	↓Albuminuria ↓Mesangial expansion ↓Fibronectin accumulation ↓Macrophage infiltration ↑O^2−^ scavenging ↑MnSOD activity ↓Mitochondrial biogenesis mRNA	[98]
Male Wistar rats	5 mg/kg/day; 16 weeks	↓Glucose levels ↓SOD activity ↓TBARS levels ↓TNF-α ↓IL-6	↓Apoptosis rate of kidney cells ↓NF-κB activity	[99]
Male Wistar rats	20 mg/kg/day; 8 weeks	↓Glucose levels ↓Creatinine levels	↓Urinary protein excretion ↓Renal hypertrophy ↓Mesangial matrix expansion ↓Mesangial cell hyperplasia ↓GSTM expression	[100]
db/db mice	20 mg/kg/day; 12 weeks	No measured effects	↓Kidney albuminuria ↓Kidney NEFA and triacylglycerol ↓Mesangial area ↓Oxidative stress ↓Type IV collagen ↓TGF-β1 ↓F4/80 positive cells ↑Ph-AMPK ↑SIRT1 protein ↓PI3K-Akt protein and activity ↓Ph-FOXO3a ↓BAX protein ↑BCL-2 production ↓Renal and Urinary 8-OHdG	[101]
FVB mice	10 mg/kg/day; 12 weeks	No measured effects	↓Glomerular area ↓Extracellular matrix ↓Albumin levels ↓Ph-Akt protein ↓PAI-1 protein ↓ICAM-1 protein ↓PCNA mRNA	[70]
Sprague–Dawley rats	200 mg/kg/day; 12 weeks	No measured effects	↓Glomerular area ↓Mesangial cell expansion ↓Glomerular basement membrane thickness ↓Collagen IV ↓Fibronectin ↑AdipoR1 expression ↓MDA production	[69]
Male Wistar rats	10 mg/kg/day; 30 days	↓Glucose levels ↓Urea nitrogen levels	↓Glomeruli sclerotic changes ↓Epithelial desquamation ↓Tissue swelling ↓Intracytoplasmic vacuolization ↓Brush border loss ↓Kidney TGF-β1 ↑SOD and CAT activities ↓MDA levels	[102]
db/db mice	40 mg/kg/day; 12 weeks	↓BUN levels ↓Creatinine levels	↓Glomerulosclerosis ↓Tubulointerstitial fibrosis ↓Albuminuria ↑Kidney SOD, Mn-SOD, Catalase protein ↓Renal MDA ↓α-SMA protein ↓E-cadherin protein ↓TGF-β, pSmad3, ph-Akt, ph-ERK ↓IGF-1R expression ↑HRD1 expression	[103]
db/db mice	20 mg/kg/day; 12 weeks	↓Triacylglycerol levels ↓NEFA levels ↑Adiponectin levels	↓Glomerular matrix expansion ↓Albuminuria ↑AdipoR1 and AdipoR2 ↑Ph-AMPK, SIRT1, total FoxO1, total FoxO3a ↑PGC-1α, ERR-1α, ph-ACC ↓SREBP-1c ↓Bax ↑Bcl-2 ↓8-OHdG levels ↓8-isoprostane levels	[104]
db/db mice	40 mg/kg/day; 12 weeks	No measured effects	↓Mesangial area ↓Albuminuria ↓Collagen deposition ↓FSP-1, α-SMA, and fibronectin protein ↓NOX4 protein ↑Ph-AMPK, ph-ACC	[94]
Sprague-Dawley rats	5 mg/kg/day; 4 months	↓Glucose levels ↓Cholesterol levels ↓Triglyceride levels ↓HbA1c levels ↓Creatinine levels ↓Urea nitrogen levels ↓Cycstatin C levels ↓TNF-a, IL-6, IL-1B, and IL-10 levels	↓Albuminuria ↓Renal 8-OHdG ↑SIRT1 mRNA and protein ↑Atg5 and Atg7 mRNA	[105]
Male Wistar rats	30 mg/kg/day; 16 weeks	↓Creatinine levels	↑Renal function ↓Kidney weight ↑Kidney SOD activity ↓Kidney MDA content ↑CAT protein ↓SIRT1 protein ↑SIRT1 activity ↓Acetylated-FOXO3a	[83]
Sprague-Dawley rats	20 mg/kg/day; 4 weeks	↓Glucose levels ↓Creatinine levels	↓Kidney weight ↓Glomerular thickening ↓Interstitial fibrosis ↓Epithelial cellular vacuolar degeneration ↓Hyaline casts ↓Arteriolopathy ↓Ph-p38 and p38 protein ↓TGF-β1 protein ↓Fibronectin protein ↓Urinary albumin	[71]
Male Wistar rats	5 mg/kg/day; 45 days	No measured effects	↓Renal hypertrophy ↓Mesangial expansion ↓Fibrosis ↓Oxidative damage ↓Kidney AGE accumulation ↓DNA damage ↓4-HNE protein ↓Caspase-3 protein ↓Cleaved caspase-3 protein	[106]
C57BL/KsJ db/+ mice	10 mg/kg/day; 8 weeks	↓Glucose levels ↓Insulin levels ↓IL-1β, IL-17, IL-10 and TNF-α levels ↑IL-6 and VEGF levels	↓Renal cell apoptosis ↓Apaf-1, caspase-3, caspase-8 and caspase-9 mRNA ↓Ph-AMPK ↓Total thiol level ↑GSH level	[107]
CD-1 mice	30 mg/kg/day; 12 weeks	↓Glucose levels ↓Cholesterol levels ↓Urea nitrogen levels	↓Glomerular thickening ↓Mesangial area ↑Podocyte mitochondria ↓Renal cell apoptosis ↑Nephrin, SIRT1, PGC-1α, NRF1, TFAM protein ↓Kidney MDA content ↓Kidney Mn-SOD activity	[87]

FFA: free-fatty acid; TBARS: thiobarbituric acid reactive substances; GSTM: glutathione S-transferase Mu; NEFA: non-esterified fatty acid; 8-OHdG: 8-hydroxydeoxyguanosine; PAI: plasminogen activator inhibitor; ICAM: intercellular adhesion molecule; PCNA: proliferating cell nuclear antigen; SOD: superoxide dismutase; Mn-SOD: manganese superoxide dismutase; BUN; blood urea nitrogen; IGF-1R: insulin-like growth factor 1 receptor; HRD1: 3-hydroxy-3-methylglutaryl reductase degradation; ERR: estrogen-related receptor; SREBP: sterol regulatory element-binding protein; FSP: fibroblast-specific protein; HbA1c: hemoglobin A1c; Atg: autophagy related; AGE: advanced glycation end production; 4-HNE: 4-Hydroxynonenal; Apaf: Apoptotic protease activating factor.

**Table 8:** Effects of resveratrol on renal fibrosis (animal studies).

Animal	Resveratrol Concentration/Duration	Serum Effects	Other Effects	Reference
Sprague–Dawley rats	10 mg/kg/day; 21 days	↓MDA levels	↓Urine calcium oxalate crystals ↓Hyaluronan protein ↓Osteopontin protein ↑GPx protein ↑CAT protein ↑SOD protein	[75]
Male Wistar rats	8 mg/kg/alternating days; 8 days	↓Creatinine levels ↓Urea nitrogen levels	↓Oxidative stress ↓Renal tubular epithelial cell necrosis ↓MDA, BUN, CRE, and ROS levels ↑SOD and GPx levels ↑Selenium content	[108]
C57BL/6J mice	20 mg/kg/day; 14 days	No measured effects	↓Extracellular matrix deposition ↓Tubulointerstitium damage ↓Oxidative stress ↓ICAM-1 mRNA ↓TNF-α mRNA ↓TGF-β mRNA ↓Acetyl-Smad3 ↓Fibronectin	[109]
UUO-Sprague-Dawley rats	20 mg/kg/day; 7–14 days	↓Creatinine levels	↓Renal interstitial damage ↓Tubular dilation and atrophy ↓Collagen deposition ↓Inflammation cell infiltration ↓α-SMA and type III collagen mRNA and protein ↑E-cadherin protein and mRNA ↓TGF-β1 expression	[77]
I/R and UUO C57BL/6 mice	20 mg/kg/day; 6 weeks	↓Creatinine levels ↓BUN levels	↑α-SMA protein ↑COL1A1 protein	[79]
Sprague–Dawley rats	50 mg/kg; 8 h	↓Creatinine levels ↓Urea nitrogen levels	↓Apoptosis ↑SIRT1 activity and protein ↑SIRT3 activity and protein ↑SOD2 protein ↓Acetyl-SOD2 ↑GSH and ATP content ↑GSH/GSSG ratio ↑CAT activity ↓mPTP opening	[110]
Male cystic (Cy/+) rats	200 mg/kg/day; 5 weeks	↓BUN levels ↓Creatinine levels	↓Cyst density ↓Macrophage infiltration ↓MCP-1 ↓TNF-α ↓CFB ↓Ph-p65, ph-S6K and p50	[81]
Sprague–Dawley rats	3 and 10 mg/kg/injection; 70 h	↓BUN levels ↓Creatinine levels ↓Nitrogen levels	↑Survival ↓Cystatin C ↓KIM-1 ↓TNF-α ↓IL-1B ↓IL-6 ↓Renal injury index	[111]
Kunming mice	10 mg/kg/day; 1 week	↓BUN levels ↓Creatinine levels	↓Apoptosis ↓Caspase-3 activity ↓Bax protein ↓ERK1/2 protein	[84]
Male AKI rats	30 mg/kg; 12 h	↓Creatinine levels ↓Urea nitrogen levels ↓TNF-α, IL-1β, IL-6 levels	↑Renal function ↓Tubular epithelial cell injury ↑Survival ↓p-65 positive cells ↓Renal TNF-α, IL-1β, IL-6 mRNA ↓IRE1 protein	[82]
5/6 Nephrectomized Sprague–Dawley rats	20 mg/kg/day; 4 weeks	No measured effects	↓Mesangial cell proliferation ↓Glomeruli matrix expansion ↓TGF-β ↑ATP production ↓ROS production ↑Activities of complex I and III ↑ATP synthase B ↑COX I, Opa1, Mfn2 ↓Drp1	[72]
C57BL/6 mice	25 and 100 mg/kg/day; 2 weeks	↓Creatinine levels	25 mg/kg RSV: ↓Renal fibrosis ↓Tubular lesion score ↓Interstitial collagen deposition ↓α-SMA protein ↓Snail protein ↓Fibronectin protein ↑SIRT1 ↓Phospho-Smad3 100 mg/kg RSV: ↑Renal fibrosis ↑α-SMA and TFAM	[85]

CRE: creatinine; GPx: glutathione peroxidase; mPTP: mitochondrial permeability transition pore; KIM-1: kidney injury molecule 1; IRE-1: Inositol-requiring enzyme 1; Opa1: optic atrophy 1; Mfn2: mitofusin 2; Drp1: dynamin related protein 1.

## References

[1] Vart P., Grams M.E. (2016). Measuring and assessing kidney function. Semin. Nephrol..

[2] Pollak M.R., Quaggin S.E., Hoenig M.P., Dworkin L.D. (2014). The glomerulus: The sphere of influence. Clin. J. Am. Soc. Nephrol..

[3] Mitrakou A. (2011). Kidney: Its impact on glucose homeostasis and hormonal regulation. Diabetes Res. Clin. Pr..

[4] Dalal R., Sehdev J.S. (2018). Physiology, renal, blood flow and filtration. StatPearls.

[5] Kurtz S.B. (1984). Renal Function: Mechanisms Preserving Fluid and Solute Balance in Health. Mayo Clin. Proc..

[6] Hansell P., Welch W.J., Blantz R.C., Palm F. (2013). Determinants of kidney oxygen consumption and their relationship to tissue oxygen tension in diabetes and hypertension. Clin. Exp. Pharmacol. Physiol..

[7] Robson L. (2014). The kidney—An organ of critical importance in physiology. J. Physiol..

[8] Schlondorff D. (1987). The glomerular mesangial cell: An expanding role for a specialized pericyte. FASEB J..

[9] Pavenstädt H. (2000). Roles of the podocyte in glomerular function. Am. J. Physiol. Physiol..

[10] Strutz F., Zeisberg M. (2006). Renal fibroblasts and myofibroblasts in chronic kidney disease. J. Am. Soc. Nephrol..

[11] Scott R.P., Quaggin S.E. (2015). The cell biology of renal filtration. J. Cell Boil..

[12] Smith P.L., Buffington D.A., Humes H.D., Klimanskaya I., Lanza R. (2006). Kidney epithelial cells. Methods in Enzymology.

[13] Subramanya A.R., Ellison D.H. (2014). Distal convoluted tubule. CJASN.

[14] Kaufman D.P., Basit H., Knohl S.J. (2019). Physiology, glomerular filtration rate (GFR). StatPearls.

[15] Musso C.G., Álvarez-Gregori J., Jáuregui J., Macías-Núñez J.F. (2016). Glomerular filtration rate equations: A comprehensive review. Int. Urol. Nephrol..

[16] Arnold R., Issar T., Krishnan A.V., A Pussell B. (2016). Neurological complications in chronic kidney disease. JRSM Cardiovasc. Dis..

[17] Lips P., Goldsmith D., De Jongh R. (2017). Vitamin D and osteoporosis in chronic kidney disease. J. Nephrol..

[18] Iorember F.M. (2018). Malnutrition in chronic kidney disease. Front Pediatr..

[19] Levin A., Hemmelgarn B., Culleton B., Tobe S., McFarlane P., Ruzicka M., Burns K., Manns B., White C., Madore F. (2008). Guidelines for the management of chronic kidney disease. Can. Med Assoc. J..

[20] Arora P., Vasa P., Brenner D., Iglar K., McFarlane P., Morrison H., Badawi A. (2013). Prevalence estimates of chronic kidney disease in Canada: Results of a nationally representative survey. Can. Med Assoc. J..

[21] Luyckx V.A., Tonelli M., Stanifer J.W. (2018). The global burden of kidney disease and the sustainable development goals. Bull. World Heal. Organ..

[22] Gajjala P.R., Sanati M., Jankowski J. (2015). Cellular and molecular mechanisms of chronic kidney disease with diabetes mellitus and cardiovascular diseases as its comorbidities. Front. Immunol..

[23] Gewin L., Zent R., Pozzi A. (2017). Progression of chronic kidney disease: too much cellular talk causes damage. Kidney Int..

[24] Zoja C., Abbate M., Remuzzi G. (2015). Progression of renal injury toward interstitial inflammation and glomerular sclerosis is dependent on abnormal protein filtration. Nephrol. Dial. Transplant..

[25] Turner J.M., Bauer C., Abramowitz M.K., Melamed M.L., Hostetter T.H. (2012). Treatment of chronic kidney disease. Kidney Int..

[26] Fraser S.D., Blakeman T., Blakeman T. (2016). Chronic kidney disease: Identification and management in primary care. Pragmatic Obs. Res..

[27] Lee-Law P.Y., Van De Laarschot L.F., Banales J.M., Drenth J.P. (2019). Genetics of polycystic liver diseases. Curr. Opin. Gastroenterol..

[28] Chadban S.J., Atkins R.C. (2005). Glomerulonephritis. Lancet.

[29] Shahbazian H., Rezaii I. (2013). Diabetic kidney disease; review of the current knowledge. J. Renal. Inj. Prev..

[30] Ferlay J., Soerjomataram I., Dikshit R., Eser S., Mathers C., Rebelo M., Parkin D.M., Forman D., Bray F. (2015). Cancer incidence and mortality worldwide: Sources, methods and major patterns in GLOBOCAN 2012. Int. J. Cancer.

[31] Siegel R.L., Miller K.D., Jemal A. (2018). Cancer statistics, 2018. CA Cancer J. Clin..

[32] Nabi S., Kessler E.R., Bernard B., Flaig T.W., Lam E.T. (2018). Renal cell carcinoma: A review of biology and pathophysiology. F1000Res..

[33] Capitanio U., Bensalah K., Bex A., Boorjian S.A., Bray F., Coleman J., Gore J.L., Sun M., Wood C., Russo P. (2019). Epidemiology of renal cell carcinoma. Eur. Urol..

[34] Barata P.C., Rini B.I. (2017). Treatment of renal cell carcinoma: Current status and future directions. CA Cancer J. Clin..

[35] Vieira A.R., Abar L., Vingeliene S., Chan D.S.M., Aune D., Navarro-Rosenblatt D., Stevens C., Greenwood D., Norat T. (2016). Fruits, vegetables and lung cancer risk: A systematic review and meta-analysis. Ann. Oncol..

[36] Kuzma J.N., Schmidt K.A., Kratz M. (2017). Prevention of metabolic diseases: Fruits (including fruit sugars) vs. vegetables. Curr. Opin. Clin. Nutr. Metab. Care.

[37] Stefan N., Häring H.-U., Schulze M.B. (2018). Metabolically healthy obesity: The low-hanging fruit in obesity treatment?. Lancet Diabetes Endocrinol..

[38] Baur J.A., Sinclair D.A. (2006). Therapeutic potential of resveratrol: the in vivo evidence. Nat. Rev. Drug Discov..

[39] Park E.-J., Pezzuto J.M. (2015). The pharmacology of resveratrol in animals and humans. Biochim. Biophys. Acta Mol. Basis Dis..

[40] Moore J., Yousef M., Tsiani E. (2016). Anticancer effects of rosemary (*Rosmarinus officinalis* L.) extract and rosemary extract polyphenols. Nutrients.

[41] Serino A., Salazar G. (2018). Protective role of polyphenols against vascular inflammation, aging and cardiovascular disease. Nutrition.

[42] Pandey K.B., Rizvi S.I. (2009). Plant polyphenols as dietary antioxidants in human health and disease. Oxid. Med. Cell. Longev..

[43] Burns J., Yokota T., Ashihara H., Lean M.E.J., Crozier A. (2002). Plant foods and herbal sources of resveratrol. J. Agric. Food Chem..

[44] Sautter C.K., DeNardin S., Alves A.O., Mallmann C.A., Penna N.G., Hecktheuer L.H. (2005). Determinação de resveratrol em sucos de uva no Brasil. Food Sci. Technol..

[45] Stervbo U., Vang O., Bonnesen C. (2007). A review of the content of the putative chemopreventive phytoalexin resveratrol in red wine. Food Chem..

[46] Prasad K. (2012). Resveratrol, wine, and atherosclerosis. Int. J. Angiol..

[47] Bo S., Ciccone G., Castiglione A., Gambino R., De Michieli F., Villois P., Durazzo M., Cavallo-Perin P., Cassader M. (2013). Anti-inflammatory and antioxidant effects of resveratrol in healthy smokers a randomized, double-blind, placebo-controlled, cross-over trial. Curr. Med. Chem..

[48] Carrizzo A., Forte M., Damato A., Trimarco V., Salzano F.A., Bartolo M., Maciąg A., Puca A.A., Vecchione C. (2013). Antioxidant effects of resveratrol in cardiovascular, cerebral and metabolic diseases. Food Chem. Toxicol..

[49] Carter L.G., D’Orazio J.A., Pearson K.J. (2014). Resveratrol and cancer: Focus on in vivo evidence. Endocr. Relat. Cancer.

[50] Wang Y., Jiang Y., Fan X., Tan H., Zeng H., Wang Y., Chen P., Huang M., Bi H. (2015). Hepato-protective effect of resveratrol against acetaminophen-induced liver injury is associated with inhibition of CYP-mediated bioactivation and regulation of SIRT1–p53 signaling pathways. Toxicol. Lett..

[51] Peiyuan H., Zhiping H., Chengjun S., Chunqing W., Bingqing L., Imam M.U. (2017). Resveratrol ameliorates experimental alcoholic liver disease by modulating oxidative stress. Evid. Based Complement. Altern. Med..

[52] Wenzel E., Somoza V. (2005). Metabolism and bioavailability of trans-resveratrol. Mol. Nutr. Food Res..

[53] Cottart C.-H., Nivet-Antoine V., Beaudeux J.-L., Laguillier-Morizot C., Laguillier-Morizot C., Nivet-Antoine V., Laguillier-Morizot C. (2010). Resveratrol bioavailability and toxicity in humans. Mol. Nutr. Food Res..

[54] Walle T. (2011). Bioavailability of resveratrol. Ann. N. Y. Acad. Sci..

[55] Boocock D.J., Faust G.E., Patel K.R., Schinas A.M., Brown V.A., Ducharme M.P., Booth T.D., Crowell J.A., Perloff M., Gescher A.J. (2007). Phase I dose escalation pharmacokinetic study in healthy volunteers of resveratrol, a potential cancer chemopreventive agent. Cancer Epidemiol. Biomark. Prev..

[56] Almeida L., Vaz-da-Silva M., Falcão A., Soares E., Costa R., Loureiro A.I., Fernandes-Lopes C., Rocha J.-F., Nunes T., Wright L. (2009). Pharmacokinetic and safety profile of trans-resveratrol in a rising multiple-dose study in healthy volunteers. Mol. Nutr. Food Res..

[57] El-Mohsen M.A., Bayele H., Kuhnle G., Gibson G., Debnam E., Srai S.K., Rice-Evans C., E Spencer J.P. (2006). Distribution of [3H]trans-resveratrol in rat tissues following oral administration. Br. J. Nutr..

[58] Juan M.E., Maijó M., Planas J.M. (2010). Quantification of trans-resveratrol and its metabolites in rat plasma and tissues by HPLC. J. Pharm. Biomed. Anal..

[59] Vallejo F., Pallarés F.J., Larrosa M., Lucas R., Morales J.C., Espín J.C., Azorín-Ortuño M., Yáñez-Gascón M.J., Tomás-Barberán F.A., García-Conesa M.T. (2011). Metabolites and tissue distribution of resveratrol in the pig. Mol. Nutr. Food Res..

[60] Burkon A., Somoza V. (2008). Quantification of free and protein-bound trans-resveratrol metabolites and identification of trans-resveratrol-C/O-conjugated diglucuronides—Two novel resveratrol metabolites in human plasma. Mol. Nutr. Food Res..

[61] Walle T., Hsieh F., DeLegge M.H., Oatis J.E. (2004). High absorption but very low bioavailability of oral resveratrol in humans. Drug Metab. Dispos..

[62] Kitching A.R., Hutton H.L. (2016). The players: Cells involved in glomerular disease. Clin. J. Am. Soc. Nephrol..

[63] Uchida Y., Yamazaki H., Watanabe S., Hayakawa K., Meng Y., Hiramatsu N., Kasai A., Yamauchi K., Yao J., Kitamura M. (2005). Enhancement of NF-kappaB activity by resveratrol in cytokine-exposed mesangial cells. Clin. Exp. Immunol..

[64] Chen F., Castranova V., Shi X., Demers L.M. (1999). New insights into the role of nuclear factor-kappaB, a ubiquitous transcription factor in the initiation of diseases. Clin. Chem..

[65] Morales A.I., Rodríguez-Barbero A., Vicente-Sánchez C., Mayoral P., Lopez-Novoa J.M., Pérez-Barriocanal F. (2006). Resveratrol inhibits gentamicin-induced mesangial cell contraction. Life Sci..

[66] Venkatesan B., Ghosh-Choudhury N., Das F., Mahimainathan L., Kamat A., Kasinath B.S., Abboud H.E., Choudhury G.G. (2008). Resveratrol inhibits PDGF receptor mitogenic signaling in mesangial cells: Role of PTP1B. FASEB J..

[67] Xu Y., Nie L., Yin Y.-G., Tang J.-L., Zhou J.-Y., Li D.-D., Zhou S.-W. (2012). Resveratrol protects against hyperglycemia-induced oxidative damage to mitochondria by activating SIRT1 in rat mesangial cells. Toxicol. Appl. Pharmacol..

[68] Zhang L., Pang S., Deng B., Qian L., Chen J., Zou J., Zheng J., Yang L., Zhang C., Chen X. (2012). High glucose induces renal mesangial cell proliferation and fibronectin expression through JNK/NF-κB/NADPH oxidase/ROS pathway, which is inhibited by resveratrol. Int. J. Biochem. Cell Boil..

[69] Ji H., Wu L., Ma X., Ma X., Qin G. (2014). The effect of resveratrol on the expression of AdipoR1 in kidneys of diabetic nephropathy. Mol. Boil. Rep..

[70] Xu F., Wang Y., Cui W., Yuan H., Sun J., Wu M., Guo Q., Kong L., Wu H., Miao L. (2014). Resveratrol prevention of diabetic nephropathy is associated with the suppression of renal inflammation and mesangial cell proliferation: Possible roles of Akt/NF-κB pathway. Int. J. Endocrinol..

[71] Qiao Y., Gao K., Wang Y., Wang X., Cui B. (2017). Resveratrol ameliorates diabetic nephropathy in rats through negative regulation of the p38 MAPK/TGF-β1 pathway. Exp. Ther. Med..

[72] Hui Y., Lu M., Han Y., Zhou H., Liu W., Li L., Jin R. (2017). Resveratrol improves mitochondrial function in the remnant kidney from 5/6 nephrectomized rats. Acta Histochem..

[73] Lee M.-J., Feliers D., Sataranatarajan K., Mariappan M.M., Li M., Barnes J.L., Choudhury G.G., Kasinath B.S. (2010). Resveratrol ameliorates high glucose-induced protein synthesis in glomerular epithelial cells. Cell. Signal..

[74] Kim D.H., Jung Y.J., Lee J.E., Lee A.S., Kang K.P., Lee S., Park S.K., Han M.K., Lee S.Y., Ramkumar K.M. (2011). SIRT1 activation by resveratrol ameliorates cisplatin-induced renal injury through deacetylation of p53. Am. J. Physiol. Renal Physiol..

[75] Hong S.H., Lee H.-J., Sohn E.J., Ko H.-S., Shim B.S., Ahn K.S., Kim S.-H. (2013). Anti-nephrolithic potential of resveratrol via inhibition of ROS, MCP-1, hyaluronan and osteopontin in vitro and in vivo. Pharmacol. Rep..

[76] Weixel K.M., Marciszyn A., Alzamora R., Li H., Fischer O., Edinger R.S., Hallows K.R., Johnson J.P. (2013). Resveratrol inhibits the epithelial sodium channel via phopshoinositides and AMP-activated protein kinase in kidney collecting duct cells. PLoS ONE.

[77] Bai Y., Lu H., Wu C., Liang Y., Wang S., Lin C., Chen B., Xia P. (2014). Resveratrol inhibits epithelial-mesenchymal transition and renal fibrosis by antagonizing the hedgehog signaling pathway. Biochem. Pharmacol..

[78] He T., Guan X., Wang S., Xiao T., Yang K., Xu X., Wang J., Zhao J. (2015). Resveratrol prevents high glucose-induced epithelial–mesenchymal transition in renal tubular epithelial cells by inhibiting NADPH oxidase/ROS/ERK pathway. Mol. Cell. Endocrinol..

[79] Xiao Z., Chen C., Meng T., Zhang W., Zhou Q. (2016). Resveratrol attenuates renal injury and fibrosis by inhibiting transforming growth factor-β pathway on matrix metalloproteinase 7. Exp. Biol. Med..

[80] Huang Y.T., Chen Y.Y., Lai Y.H., Cheng C.C., Lin T.C., Su Y.S., Liu C.H., Lai P.C. (2016). Resveratrol alleviates the cytotoxicity induced by the radiocontrast agent, ioxitalamate, by reducing the production of reactive oxygen species in HK-2 human renal proximal tubule epithelial cells in vitro. Int. J. Mol. Med..

[81] Gu J., Mei S., Xu D., Chen M., Chen S., Hu H., Mei C., Wu M., Jing Y., Yao Q. (2016). Resveratrol delays polycystic kidney disease progression through attenuation of nuclear factor κB-induced inflammation. Nephrol. Dial. Transplant..

[82] Wang N., Mao L., Yang L., Zou J., Liu K., Liu M., Zhang H., Xiao X., Wang K. (2017). Resveratrol protects against early polymicrobial sepsis-induced acute kidney injury through inhibiting endoplasmic reticulum stress-activated NF-κB pathway. Oncotarget.

[83] Wang X., Meng L., Zhao L., Wang Z., Liu H., Liu G., Guan G. (2017). Resveratrol ameliorates hyperglycemia-induced renal tubular oxidative stress damage via modulating the SIRT1/FOXO3a pathway. Diabetes Res. Clin. Pract..

[84] Fu B., Zhao J., Peng W., Wu H., Zhang Y. (2017). Resveratrol rescues cadmium-induced mitochondrial injury by enhancing transcriptional regulation of PGC-1α and SOD2 via the Sirt3/FoxO3a pathway in TCMK-1 cells. Biochem. Biophys. Res. Commun..

[85] Liu S., Zhao M., Zhou Y., Wang C., Yuan Y., Li L., Bresette W., Chen Y., Cheng J., Lu Y. (2018). Resveratrol exerts dose-dependent anti-fibrotic or pro-fibrotic effects in kidneys: A potential risk to individuals with impaired kidney function. Phytomedicine.

[86] Yang R.-C., Zhu X.-L., Zhang H.-Q., Li W.-D. (2013). Study of resveratrol suppressing TGF-beta1 induced transdifferentiation of podocytes. Chin. J. Integr. Tradit. West. Med..

[87] Zhang T., Chi Y., Kang Y., Lu H., Niu H., Liu W., Li Y. (2019). Resveratrol ameliorates podocyte damage in diabetic mice via SIRT1/PGC-1α mediated attenuation of mitochondrial oxidative stress. J. Cell. Physiol..

[88] Lechner M.S., Dressler G.R. (1997). The molecular basis of embryonic kidney development. Mech. Dev..

[89] Lin Y.-C., Boone M., Meuris L., Lemmens I., Van Roy N., Soete A., Reumers J., Moisse M., Plaisance S., Drmanac R. (2014). Genome dynamics of the human embryonic kidney 293 lineage in response to cell biology manipulations. Nat. Commun..

[90] Rössler O.G., Glatzel D., Thiel G. (2015). Resveratrol upregulates Egr-1 expression and activity involving extracellular signal-regulated protein kinase and ternary complex factors. Exp. Cell Res..

[91] Bui-Klimke T.R., Wu F. (2015). Ochratoxin A and human health risk: A review of the evidence. Crit. Rev. Food Sci. Nutr..

[92] Raghubeer S., Nagiah S., Phulukdaree A., Chuturgoon A. (2015). The phytoalexin resveratrol ameliorates ochratoxin a toxicity in human embryonic kidney (HEK293) cells. J. Cell. Biochem..

[93] Abharzanjani F., Afshar M., Hemmati M., Moossavi M. (2017). Short-term high dose of quercetin and resveratrol alters aging markers in human kidney cells. Int. J. Prev. Med..

[94] He T., Xiong J., Nie L., Yu Y., Guan X., Xu X., Xiao T., Yang K., Liu L., Zhang D. (2016). Resveratrol inhibits renal interstitial fibrosis in diabetic nephropathy by regulating AMPK/NOX4/ROS pathway. J. Mol. Med..

[95] Zhang H., Yang R., Zhu L. (2011). Inhibitory effect of resveratrol on the expression of the VEGF gene and proliferation in renal cancer cells. Mol. Med. Rep..

[96] Kim C., Baek S.H., Um J.-Y., Shim B.S., Ahn K.S. (2016). Resveratrol attenuates constitutive STAT3 and STAT5 activation through induction of PTPε and SHP-2 tyrosine phosphatases and potentiates sorafenib-induced apoptosis in renal cell carcinoma. BMC Nephrol..

[97] Zhao Y., Tang H., Zeng X., Ye D., Liu J. (2018). Resveratrol inhibits proliferation, migration and invasion via Akt and ERK1/2 signaling pathways in renal cell carcinoma cells. Biomed. Pharmacother..

[98] Kitada M., Kume S., Imaizumi N., Koya D. (2011). Resveratrol improves oxidative stress and protects against diabetic nephropathy through normalization of Mn-SOD dysfunction in AMPK/SIRT1-independent pathway. Diabetes.

[99] Soufi F.G., Vardyani M., Sheervalilou R., Mohammadi M., Somi M.H. (2012). Long-term treatment with resveratrol attenuates oxidative stress pro-inflammatory mediators and apoptosis in streptozotocin-nicotinamide-induced diabetic rats. Gen. Physiol. Biophys..

[100] Jiang B., Guo L., Li B.-Y., Zhen J.-H., Song J., Peng T., Yang X.-D., Hu Z., Gao H.-Q. (2013). Resveratrol attenuates early diabetic nephropathy by down-regulating glutathione s-transferases mu in diabetic rats. J. Med. Food.

[101] Kim M.Y., Lim J.H., Youn H.H., Hong Y.A., Yang K.S., Park H.S., Chung S., Ko S.H., Koh S.H., Shin S.J. (2013). Resveratrol prevents renal lipotoxicity and inhibits mesangial cell glucotoxicity in a manner dependent on the AMPK-SIRT1-PGC1α axis in db/db mice. Diabetologia.

[102] Elbe H., Vardi N., Esrefoglu M., Ates B., Yologlu S., Taskapan C. (2015). Amelioration of streptozotocin-induced diabetic nephropathy by melatonin, quercetin, and resveratrol in rats. Hum. Exp. Toxicol..

[103] Yan C., Xu W., Huang Y., Li M., Shen Y., You H., Liang X. (2016). HRD1-mediated IGF-1R ubiquitination contributes to renal protection of resveratrol in db/db mice. Mol. Endocrinol..

[104] Park H.S., Lim J.H., Kim M.Y., Kim Y., Hong Y.A., Choi S.R., Chung S., Kim H.W., Choi B.S., Kim Y.S. (2016). Resveratrol increases AdipoR1 and AdipoR2 expression in type 2 diabetic nephropathy. J. Transl. Med..

[105] Ma L., Fu R., Duan Z., Lu J., Gao J., Tian L., Lv Z., Chen Z., Han J., Jia L. (2016). Sirt1 is essential for resveratrol enhancement of hypoxia-induced autophagy in the type 2 diabetic nephropathy rat. Pathol. Res. Pr..

[106] Al-Hussaini H., Kilarkaje N. (2018). Trans-resveratrol mitigates type 1 diabetes-induced oxidative DNA damage and accumulation of advanced glycation end products in glomeruli and tubules of rat kidneys. Toxicol. Appl. Pharmacol..

[107] Guo H., Zhang L. (2018). Resveratrol provides benefits in mice with type II diabetes-induced chronic renal failure through AMPK signaling pathway. Exp. Ther. Med..

[108] Zhang W., Liu Y., Ge M., Jing J., Chen Y., Jiang H., Yu H., Li N., Zhang Z. (2014). Protective effect of resveratrol on arsenic trioxide-induced nephrotoxicity in rats. Nutr. Res. Pr..

[109] Liang J., Tian S., Han J., Xiong P. (2014). Resveratrol as a therapeutic agent for renal fibrosis induced by unilateral ureteral obstruction. Ren. Fail..

[110] Xu S., Gao Y., Zhang Q., Wei S., Chen Z., Dai X., Zeng Z., Zhao K.-S. (2016). SIRT1/3 activation by resveratrol attenuates acute kidney injury in a septic rat model. Oxid. Med. Cell Longev..

[111] Gan Y., Tao S., Cao D., Xie H., Zeng Q. (2017). Protection of resveratrol on acute kidney injury in septic rats. Hum. Exp. Toxicol..

[112] Saldanha J.F., Leal V.O., Rizzetto F., Grimmer G.H., Ribeiro-Alves M., Daleprane J.B., Carraro-Eduardo J.C., Mafra D. (2016). Effects of resveratrol supplementation in Nrf2 and NF-κB expressions in nondialyzed chronic kidney disease patients: A randomized, double-blind, placebo-controlled, crossover clinical trial. J. Ren. Nutr..

[113] Lin C.-T., Sun X.-Y., Lin A.-X. (2016). Supplementation with high-dose trans-resveratrol improves ultrafiltration in peritoneal dialysis patients: a prospective, randomized, double-blind study. Ren. Fail..

[114] Saldanha J.F., Leal V.D.O., Stenvinkel P., Carraro-Eduardo J.C., Mafra D. (2013). Resveratrol: Why is it a promising therapy for chronic kidney disease patients?. Oxid. Med. Cell Longev..

[115] Singh A.P., Singh R., Verma S.S., Rai V., Kaschula C.H., Maiti P., Gupta S.C. (2019). Health benefits of resveratrol: Evidence from clinical studies. Med. Res. Rev..

[116] Brasnyó P., Molnár G.A., Mohás M., Markó L., Laczy B., Cseh J., Mikolás E., Szijártó I.A., Mérei A., Halmai R. (2011). Resveratrol improves insulin sensitivity, reduces oxidative stress and activates the akt pathway in type 2 diabetic patients. Br. J. Nutr..

[117] Bhatt J.K., Thomas S., Nanjan M.J. (2012). Resveratrol supplementation improves glycemic control in type 2 diabetes mellitus. Nutr. Res..

